# A Novel Entropy-Based EWMA Monitoring Scheme for Detecting Information Degradation Under Progressive Type-II Censoring

**DOI:** 10.3390/e28090979

**Published:** 2026-09-02

**Authors:** Ayse Bugatekin

**Affiliations:** Department of Statistics, Faculty of Science, Fırat University, 23119 Elazig, Turkey; aturan@firat.edu.tr

**Keywords:** Entropy–EWMA, progressive type-II censoring, information degradation, exponentiated Weibull distribution

## Abstract

Conventional statistical process monitoring approaches primarily focus on changes in location, dispersion, or distributional characteristics. However, process deterioration may also emerge through variations in uncertainty and information structure. Motivated by this limitation, this study proposes an Entropy–EWMA monitoring framework for progressively Type-II censored lifetime data. Developed under the Exponentiated Weibull distribution, the framework incorporates a censoring-adjusted entropy estimator into an adaptive EWMA structure to account for information loss and monitor changes in process uncertainty. The statistical performance of the proposed approach is evaluated through Monte Carlo simulations under different entropy degradation levels, censoring structures, observed sample sizes, and auxiliary shift-estimation smoothing parameter values. Control limits are calibrated to achieve an in-control Average Run Length close to the nominal target of $ARL_{0}=370$. The results show satisfactory in-control performance and substantially decreasing out-of-control Average Run Length as entropy degradation increases. Comparative simulations further demonstrate shorter run lengths for the Entropy–EWMA scheme under moderate and severe entropy degradation relative to the Classical EWMA. The practical applicability of the method is illustrated using Wind Turbine SCADA data under Uniform and Late Progressive Type-II censoring schemes. Neither method produces false alarms during the in-control phase, while both detect changes during the out-of-control phase. The Classical EWMA provides earlier and more persistent signals associated with location changes, whereas the Entropy–EWMA responds to changes in process uncertainty. These findings demonstrate that the two approaches capture different aspects of process behavior and can be interpreted as complementary monitoring tools.

## 1. Introduction

Statistical process monitoring (SPM) plays a fundamental role in modern quality control, reliability engineering, and industrial decision-making by enabling early detection of process deterioration and abnormal system behavior. Classical statistical process control (SPC) techniques were originally developed to identify departures from stable operating conditions through graphical monitoring procedures. Among these methods, the exponentially weighted moving average (EWMA) control chart introduced by [1] has become one of the most influential monitoring tools due to its ability to detect small and persistent process shifts. Subsequent developments by [2,3] and later summarized comprehensively by [4] established EWMA-based monitoring as a standard methodology in industrial and reliability applications.

Conventional monitoring approaches primarily focus on detecting shifts in process location, dispersion, percentile structure, or parameter values. Although these methods have demonstrated strong performance under complete observations, process deterioration is not necessarily reflected only through changes in central tendency. In many real engineering systems, degradation may emerge through changes in uncertainty, information content, and hidden structural variability before significant changes appear in conventional process statistics. Therefore, monitoring frameworks based solely on location or quantile changes may fail to detect early-stage information degradation.

Information theory provides a natural framework for quantifying uncertainty and structural changes in stochastic systems. The concept of entropy introduced by Shannon [5] established a mathematical measure of uncertainty and later became an important analytical tool in reliability analysis, survival modeling, and statistical inference. Cover and Thomas [6] further extended information-theoretic foundations and demonstrated broad applicability across engineering and statistical domains. Entropy-based approaches have increasingly attracted attention in survival and reliability studies due to their ability to capture latent uncertainty beyond conventional distributional measures. Important contributions include cumulative residual entropy and survival-based uncertainty measures proposed in the reliability literature, showing that entropy can provide additional information regarding process degradation mechanisms.

In reliability engineering and lifetime analysis, complete observations are often unavailable due to economic limitations, inspection schedules, maintenance interruptions, or experimental constraints. Consequently, censoring mechanisms become an unavoidable component of practical data collection. Among existing censoring structures, Progressive Type-II censoring has received considerable attention because it offers operational flexibility while preserving experimental efficiency. The theoretical foundations established by Balakrishnan and Aggarwala [7], later expanded by Balakrishnan [8], Kundu and Joarder [9], and Meeker and Escobar [10], demonstrated that progressively censored experiments provide realistic representations of industrial lifetime testing environments.

Recent developments have extended monitoring methodologies toward censored reliability settings. Wang et al. [11] proposed control charts for monitoring Weibull percentiles under complete and Type-II censored observations. Yu et al. [12] investigated monitoring of Weibull scale parameters under right-censored observations. More recently, Atif et al. [13] developed an adaptive quantile monitoring framework under censoring and demonstrated that monitoring based on distributional functionals can substantially improve detection capability. Additional studies on progressive censoring and reliability inference have been reported by Alsarray et al. [14], Alam and Nassar [15], Ren and Hu [16], El-Sherpieny et al. [17], and related recent works. Despite these developments, existing monitoring frameworks predominantly concentrate on parameter estimation, percentile monitoring, or quantile-based control structures. In addition, considerable attention has been devoted in recent years to monitoring methodologies and reliability analysis under censored observations, reflecting the growing importance of this research area [18,19,20,21,22,23].

However, studies directly integrating entropy-based information monitoring with EWMA structures under Progressive Type-II censoring remain extremely limited. In particular, little attention has been given to monitoring information degradation in censored lifetime processes where uncertainty itself becomes the primary indicator of process deterioration. This limitation motivates the development of a monitoring framework capable of detecting changes in process information rather than solely tracking classical location or scale shifts.

To model censored lifetime observations, this study adopts the Exponentiated Weibull distribution (EWD) as the baseline lifetime model. The Exponentiated Weibull distribution represents a flexible extension of the classical Weibull distribution and has been widely employed in reliability engineering and survival analysis. Unlike the standard Weibull model, which generally accommodates increasing, decreasing, or constant hazard behavior, the Exponentiated Weibull distribution introduces an additional shape parameter that substantially expands modeling flexibility. As demonstrated by Mudholkar and Srivastava [24], Mudholkar et al. [25], Nassar and Eissa [26], Pal et al. [27], and more recently Ishag et al. [28], the distribution can successfully represent increasing, decreasing, bathtub-shaped, and unimodal hazard structures. This flexibility makes the Exponentiated Weibull distribution particularly suitable for reliability systems exhibiting multiple degradation mechanisms, early failures, random deterioration, and aging effects.

Motivated by these observations, this study proposes a novel Entropy–EWMA monitoring framework for progressively Type-II censored lifetime data. The proposed approach monitors changes in information structure through an entropy-based monitoring statistic derived under the Exponentiated Weibull model and integrates this information into an adaptive EWMA mechanism. Unlike existing quantile- or parameter-oriented approaches, the proposed framework directly targets uncertainty evolution and information degradation.

The main contributions of this study are summarized as follows:

- A new entropy-based EWMA monitoring statistic is developed for progressively Type-II censored lifetime observations.
- An adjusted entropy estimation framework is introduced to account for information loss under censoring.
- The Exponentiated Weibull distribution is incorporated to provide flexible modeling of complex lifetime behaviors.
- Extensive simulation experiments are conducted to evaluate monitoring performance under different censoring structures, entropy degradation levels, and smoothing parameters.
- A real industrial application based on Wind Turbine SCADA data is presented to demonstrate practical applicability.

The remainder of this paper is organized as follows. Section 2 presents the proposed entropy-based monitoring methodology and censoring framework. Section 3 introduces the monitoring algorithm and control limits. Section 4 reports simulation results and performance evaluation. Section 5 presents the real-data application using Wind Turbine SCADA observations. Finally, Section 6 concludes the study and outlines possible directions for future research.

## 2. Statistical Framework

The proposed entropy-based monitoring framework is not inherently restricted to the Exponentiated Weibull distribution. More generally, an exponentiated family can be constructed from an arbitrary continuous baseline lifetime distribution. Let $F\left(x;\theta \right)$ and $f\left(x;\theta \right)$ denote the cumulative distribution function and probability density function of a continuous baseline lifetime model, respectively. A flexible exponentiated family can be generated through

$$G\left(x;\theta ,\gamma \right)={F\left(x;\theta \right)}^{\gamma }\;,\;\;\;\gamma >0$$

with the corresponding density

$$g\left(x;\theta ,\gamma \right)=\gamma {f\left(x;\theta \right)F\left(x;\theta \right)}^{\gamma -1}.$$

Thus, the entropy-based monitoring principle developed in this study can, in principle, be formulated for a broad class of continuous lifetime distributions, provided that the corresponding density and entropy measure can be evaluated. This representation also shows that exponentiation provides a general mechanism for introducing additional shape flexibility into a baseline lifetime model.

Within this general class, the Exponentiated Weibull distribution is adopted in the present study as a representative lifetime model. This choice is not a mathematical requirement of the proposed monitoring scheme. Rather, the Exponentiated Weibull model extends the classical Weibull distribution through an additional shape parameter and is capable of representing a broad range of distributional and hazard-rate behaviors. Such flexibility is particularly useful for the present purpose because entropy summarizes the overall uncertainty of the lifetime distribution and may respond to simultaneous changes in shape, dispersion, and tail behavior that are not necessarily reflected by a comparable change in the process mean or in a single selected quantile. Consequently, the Exponentiated Weibull distribution provides a flexible setting for developing and evaluating the proposed Entropy–EWMA methodology, while the underlying monitoring principle remains applicable to other suitable continuous lifetime models.

### 2.1. Exponentiated Weibull Distribution

The Exponentiated Weibull distribution can be viewed as a particular member of the general exponentiated family of distributions. Let $G\left(x;\theta \right)$ denote the cumulative distribution function of an arbitrary continuous baseline distribution with corresponding density $g\left(x;\theta \right)$. An exponentiated family is obtained by introducing an additional shape parameter α>0, such that $F\left(x\right)={\left[G\left(x;\theta \right)\right]}^{\alpha }$ and $f\left(x\right)=\alpha g\left(x;\theta \right){\left[G\left(x;\theta \right)\right]}^{\alpha -1}$. This formulation shows that the exponentiation mechanism is not restricted to the Weibull distribution and can be applied to other continuous baseline models. In the present study, the Weibull distribution is adopted as the baseline because its exponentiated extension provides substantial flexibility in lifetime and hazard-rate behavior while retaining a tractable structure for likelihood-based estimation and entropy evaluation under progressive censoring.

If the random variable *X* follows the Exponentiated Weibull distribution, then its probability density function (PDF) and cumulative distribution function (CDF) are, respectively, given by

(1) $$f\left(x;\alpha ,\beta ,\lambda \right)=\alpha \frac{\beta }{\lambda }{\left(\frac{x}{\lambda }\right)}^{\beta -1}ex{p\left[-{\left(\frac{x}{\lambda }\right)}^{\beta }\right]\left[1-exp\left(-{\left(\frac{x}{\lambda }\right)}^{\beta }\right)\right]}^{\alpha -1},x>0;\alpha ,\beta ,\lambda >0$$

and

(2) $$F\left(x;\alpha ,\beta ,\lambda \right)={\left[1-exp\left(-{\left(\frac{x}{\lambda }\right)}^{\beta }\right)\right]}^{\alpha }$$

[24,26,27]. Here, α and β denote the shape parameters, while λ represents the scale parameter. It is noteworthy that the Exponentiated Weibull distribution reduces to the classical Weibull distribution when α=1.

In general, the reliability (or survival) function of a continuous lifetime distribution is defined as

$$R\left(x\right)=P\left(X>x\right)=1-F(x)$$

where F(x) denotes the cumulative distribution function. For the Exponentiated Weibull distribution, the corresponding reliability function is obtained as follows.

The corresponding reliability function (RF) and hazard rate function (HRF) are obtained as

$$R\left(x;\alpha ,\beta ,\lambda \right)={1-\left[1-exp\left(-{\left(\frac{x}{\lambda }\right)}^{\beta }\right)\right]}^{\alpha }$$

and

$$h\left(x;\alpha ,\beta ,\lambda \right)=\frac{\alpha \frac{\beta }{\lambda }{\left(\frac{x}{\lambda }\right)}^{\beta -1}ex{p\left[-{\left(\frac{x}{\lambda }\right)}^{\beta }\right]\left[1-exp\left(-{\left(\frac{x}{\lambda }\right)}^{\beta }\right)\right]}^{\alpha -1}}{1-{\left[1-exp\left(-{\left(\frac{x}{\lambda }\right)}^{\beta }\right)\right]}^{\alpha }}$$

One of the major advantages of the Exponentiated Weibull distribution is its ability to generate increasing, decreasing, bathtub-shaped, and unimodal hazard structures depending on the parameter configurations. This flexibility makes the distribution particularly suitable for modeling latent degradation mechanisms and complex reliability behaviors observed in modern engineering systems.

Furthermore, the quantile function (QF), obtained as the inverse of the cumulative distribution function, can be expressed as

$$Q\left(u\right)=x_{u}=F^{-1}\left(u\right),$$

$$Q\left(u\right)=\lambda {\left[-\log\left(1-u^{\frac{1}{\alpha }}\right)\right]}^{\frac{1}{\beta }},\;0<u<1$$

The corresponding median of the distribution is therefore given by

$$Q\left(\frac{1}{2}\right)=x_{median}=F^{-1}\left(\frac{1}{2}\right)$$

$$Q\left(\frac{1}{2}\right)=\lambda {\left[-log(1-0.5^{\frac{1}{\alpha }})\right]}^{1/\beta }$$

### 2.2. Progressive Type-II Censoring

Progressive Type-II censoring (PT-II censoring) is one of the most flexible and widely used censoring mechanisms in reliability engineering, degradation analysis, and industrial lifetime monitoring studies. In real engineering systems, it is often impractical to continuously observe all operating units until complete failure. During the monitoring process, some units may be removed from the system due to maintenance operations, calibration procedures, cost limitations, data acquisition problems, or operational requirements. Consequently, incomplete or censored lifetime observations naturally arise [13].

Particularly in complex engineering applications such as turbofan engine systems, some engines may be withdrawn from the monitoring procedure before complete failure because of preventive maintenance schedules, sensor malfunctions, communication interruptions, or operational inspections. Therefore, PT-II censoring provides a highly realistic framework for modeling incomplete lifetime observations encountered in practical engineering environments.

Suppose that *n* engines are continuously monitored during the experiment, and only m<n complete failure times are eventually observed. Let $R_{1},\;R_{2},\ldots ,R_{m}$ denote the pre-specified censoring scheme, where $R_{i}$ represents the number of functioning engines removed immediately after observing the i*th* failure time. When the first failure time $X_{1:m:n}$ is observed, $R_{1}$ engines are randomly withdrawn from the experiment. After observing the second failure time $X_{2:m:n}$, another $R_{2}$ engines are removed, and this process continues until the m*th* observed failure time $X_{m:m:n}$. After the final observed failure, all remaining units are considered censored. The total number of monitored units satisfies

$$n=m+\sum_{i=1}^{m}R_{i}$$

Traditional Type-II censoring is obtained as a special case when censoring occurs only at the end of the experiment, namely,

$$R_{1}=R_{2}=\cdots =R_{m-1}=0,\;R_{m}=n-m$$

Moreover, when

$$R_{1}=R_{2}=\cdots =R_{m}=0$$

the scheme reduces to a complete sample.

The PT-II censoring mechanism provides a natural representation of real engineering operations. For instance, some engines may be removed from the monitoring process due to maintenance scheduling, safety inspections, or sensor-related issues before the experiment is fully completed. Consequently, PT-II censoring offers a realistic and effective framework for reliability analysis and lifetime modeling under operational constraints.

In this study, the probabilistic structure of censored lifetime observations is modeled under the Exponentiated Weibull distribution. Let f(.) and F(.) denote the probability density function and cumulative distribution function of the underlying population, respectively. Then, the likelihood function for a PT-II censored sample of size *m* can be expressed as

(3) $$L=C\prod_{i=1}^{m}f\left(x_{i:m:n}\right)[1{-F\left(x_{i:m:n}\right)]}^{R_{i}}$$

where $C=n\left(n-R_{1}-1\right)\ldots (n-R_{1}-R_{2}-\cdots -R_{m-1}-m+1)$ is a constant independent of the model parameters [7].

The primary objective of the proposed Entropy-EWMA monitoring framework is to monitor distortions in the information structure and uncertainty dynamics of progressively censored lifetime processes. During the censoring procedure, the systematic removal of observations reduces the available information content of the lifetime data and leads to what may be interpreted as information degradation within the monitored process. Therefore, entropy-based monitoring structures provide an effective mechanism for the early detection of latent degradation patterns and uncertainty shifts arising in censored lifetime systems.

### 2.3. Maximum Likelihood Estimation and Adjusted Entropy Under Progressive Type-II Censoring

Let $x=(x_{1:m:n},x_{2:m:n},\ldots ,x_{m:m:n})$ denote a Progressive Type-II censored sample of size *m* obtained from a lifetime experiment following the Exponentiated Weibull distribution. For convenience, let $x_{i}{=x}_{i:m:n},\;i=1,2,\ldots ,m$. Using the general likelihood structure given in Equation (3), the likelihood function for the Exponentiated Weibull distribution under Progressive Type-II censoring can be written as

$$L\left(\alpha ,\beta ,\lambda :x\right)=C\prod_{i=1}^{m}f\left(x_{i};\alpha ,\beta ,\lambda \right)[1{-F\left(x_{i};\alpha ,\beta ,\lambda \right)]}^{R_{i}}$$

where C is a constant independent of the model parameters. Substituting the probability density and cumulative distribution functions of the Exponentiated Weibull distribution into the above expression yields

$$L\left(x;\alpha ,\beta ,\lambda \right)\propto {\left(\alpha \beta \right)}^{m}\lambda^{-m\beta }\prod_{i=1}^{m}x_{i}^{\beta -1}exp\left[-\sum_{i=1}^{m}{\left(\frac{x_{i}}{\lambda }\right)}^{\beta }\right].\prod_{i=1}^{m}{\left[1-exp\left(-{\left(\frac{x_{i}}{\lambda }\right)}^{\beta }\right)\right]}^{\alpha -1}\;.\prod_{i=1}^{m}{\left\{{1-\left[1-exp\left(-{\left(\frac{x_{i}}{\lambda }\right)}^{\beta }\right)\right]}^{\alpha }\right\}}^{R_{i}}$$

Taking the natural logarithm of the likelihood function gives the log-likelihood function

$$l\left(\alpha ,\beta ,\lambda \right)=logL\left(x;\alpha ,\beta ,\lambda \right)$$

which can be expressed as

$$l\left(\alpha ,\beta ,\lambda \right)=mlog\alpha +mlog\beta -m\beta log\lambda +(\beta -1)\sum_{i=1}^{m}logx_{i}\;-\sum_{i=1}^{m}{\left(\frac{x_{i}}{\lambda }\right)}^{\beta }+\left(\alpha -1\right)\sum_{i=1}^{m}log\left[1-exp\left(-{\left(\frac{x_{i}}{\lambda }\right)}^{\beta }\right)\right]\;+\sum_{i=1}^{m}R_{i}log\left\{{1-\left[1-exp\left(-{\left(\frac{x_{i}}{\lambda }\right)}^{\beta }\right)\right]}^{\alpha }\right\}$$

The maximum likelihood estimators of the parameters α, β, and λ, denoted, respectively, by $\hat{\alpha }$, $\hat{\beta }$, and $\hat{\lambda }$, can be obtained by maximizing the log-likelihood function or equivalently by solving the associated nonlinear likelihood equations simultaneously. Since closed-form solutions are generally unavailable, the estimators can be computed numerically using iterative optimization procedures such as the Newton–Raphson algorithm.

Unlike conventional monitoring approaches based on quantile estimation, the proposed framework focuses on monitoring changes in the information structure of progressively censored lifetime observations. For this purpose, an entropy-based monitoring statistic is constructed using the estimated Exponentiated Weibull density function. For a continuous random variable, Shannon differential entropy is a fundamental information-theoretic measure that quantifies the average information content associated with the probability density function and is defined as

$$H\left(X\right)=-E\left[logf\left(X;\theta \right)\right].$$

For a complete random sample of size *n*, a natural sample analogue of the entropy is

$${\hat{H}}_{complete}=-\frac{1}{n}\sum_{i=1}^{n}logf(x_{i};\theta )$$

Under Progressive Type-II censoring, however, only m<n failure times are observed, while the remaining units are progressively removed from the experiment according to a prespecified censoring scheme. Consequently, the complete-data sample average cannot be directly applied to the observed censored sample without accounting for the information represented by the progressively removed units. To accommodate this censoring structure, a weighted entropy estimator is therefore introduced.

Let t=1,2,… denote the monitoring epoch, where a new progressively Type-II censored sample is observed at each epoch. For the t*th* censored sample, the Exponentiated Weibull parameters are estimated by maximum likelihood, yielding

$${\hat{\theta }}_{t}={\left({\hat{\alpha }}_{t},{\hat{\beta }}_{t},{\hat{\lambda }}_{t}\right)}^{T}$$

Thus, the parameter estimates are sample-specific and are updated at each monitoring epoch rather than being fixed throughout the monitoring process. The adjusted entropy statistic at epoch *t* is subsequently computed by substituting these current parameter estimates into the censoring-adjusted entropy estimator. Accordingly, the proposed adjusted entropy estimator is defined as

$${\hat{H}}_{t}^{adj}=-\sum_{i=1}^{m}w_{i}logf\left(x_{\left(i,t\right)};{\hat{\alpha }}_{t},{\hat{\beta }}_{t},{\hat{\lambda }}_{t}\right),$$

where $w_{i}$ represents the normalized stage-representation weight associated with the i*th* observed failure time.

Although the individual MLEs vary across monitoring epochs and could themselves be monitored, the objective of the proposed framework is fundamentally different. Monitoring ${\hat{\alpha }}_{t},{\hat{\beta }}_{t}$ and ${\hat{\lambda }}_{t}$ separately is primarily designed to detect parameter-specific changes and requires prior identification of which parameter, or combination of parameters, is relevant to process deterioration. Similarly, monitoring the mean or a particular quantile focuses on a specific distributional feature and may not fully reflect changes occurring simultaneously in other aspects of the lifetime distribution. Entropy is particularly useful in this context because it provides a single distribution-level measure of uncertainty that depends on the overall probability structure rather than on one selected location, scale, or quantile characteristic. Consequently, changes in shape, scale, dispersion, or tail behavior can be reflected through their combined effect on entropy, even when the corresponding change in an individual parameter or summary measure is relatively limited. This property is especially relevant under Progressive Type-II censoring, where only part of the lifetime information is directly observed. Therefore, the proposed Entropy–EWMA chart should not be viewed as a replacement for parameter-, mean-, or quantile-based monitoring. Instead, it provides a complementary information-oriented monitoring mechanism designed to detect changes in the overall uncertainty structure of the lifetime process.

In this study, the weights are defined as

$$w_{i}=\frac{1+R_{i}}{n},$$

where $R_{i}$ denotes the number of surviving units removed immediately after the i*th* observed failure and *n* denotes the initial number of units placed on the test. This weighting scheme follows directly from the structure of Progressive Type-II censoring. At the i*th* censoring stage, one failure is observed and $R_{i}$ additional surviving units are removed from the experiment. Thus, $1+R_{i}$ represents the number of units associated with the i*th* censoring stage, and division by n expresses this quantity as a relative contribution to the original sample. Since

$$\sum_{i=1}^{m}\left({1+R}_{i}\right)=m+\sum_{i=1}^{m}R_{i}=n$$

the proposed weights satisfy

$$\sum_{i=1}^{m}w_{i}=1.$$

Accordingly, $w_{i}$ can be interpreted as a normalized stage-representation weight, so that censoring stages at which more units leave the experiment receive proportionally greater representation in the adjusted entropy statistic. Importantly, these weights are determined entirely by the prespecified censoring scheme and are not estimated from the observed failure times or from the maximum likelihood estimates obtained at monitoring epoch *t*.

Consequently, unlike an estimator normalised by 1/n over only m terms, the proposed weighted estimator is asymptotically consistent with Shannon differential entropy as the sample grows, since the weights represent the full probability mass of the progressive censoring scheme.

The proposed formulation is also consistent with the complete-data case. When no censoring is present, $R_{i}=0$ for all *i* and m=n, so that $w_{i}=1/n$. Consequently, the adjusted entropy estimator reduces to

$${\hat{H}}_{t}^{adj}=-\frac{1}{n}\sum_{i=1}^{n}logf\left(x_{\left(i,t\right)};{\hat{\alpha }}_{t},{\hat{\beta }}_{t},{\hat{\lambda }}_{t}\right),$$

which coincides with the complete-sample entropy estimator defined above.

Under the standard Progressive Type-II censoring mechanism considered in this study, the removal numbers $R_{1},R_{2}$,…,$\;R_{m}$ are specified in advance by the censoring scheme. Immediately after the i*th* failure is observed, $R_{i}$ units are randomly withdrawn from the surviving units that remain at risk. The particular units to be removed are therefore not selected according to their observed or latent lifetimes. The removal mechanism is assumed to be non-informative and independent of the underlying lifetime process. Thus, the censoring scheme determines how many units are removed at each failure time, whereas the particular surviving units to be withdrawn are selected at random.

The proposed adjusted entropy statistic can therefore be interpreted as a weighted information-content measure for progressively censored lifetime processes. Larger deviations in the entropy structure indicate changes in the overall uncertainty structure of the monitored system and may provide evidence of latent process degradation. To establish the in-control reference distribution, let *D* denote the number of independent Phase I samples and let ${\hat{H}}_{d}^{adj}$ denote the adjusted entropy estimate obtained from the d*th* sample. The *D* samples are used only during Phase I calibration to estimate the in-control reference quantities ${\hat{\mu }}_{H}$ and ${\hat{\sigma }}_{H}$; they do not represent repeated sampling at each Phase II monitoring epoch. Once these reference quantities have been established, only the current progressively censored sample is required to compute ${\hat{H}}_{t}^{adj}$ at monitoring epoch *t*. The mean and standard deviation of the adjusted entropy estimator are then computed as

$${\hat{\mu }}_{H}=\frac{1}{D}\sum_{d=1}^{D}{\hat{H}}_{d}^{adj},$$

and

$${\hat{\sigma }}_{H}=\sqrt{\frac{1}{D}\sum_{d=1}^{D}{\left({\hat{H}}_{d}^{adj}-{\hat{\mu }}_{H}\right)}^{2}}.$$

These quantities are subsequently used to standardize the monitoring statistic within the proposed Entropy–EWMA control chart framework. Consequently, rather than monitoring only a specific distributional characteristic, such as a location parameter or a selected quantile, the proposed methodology is designed to monitor changes in the overall uncertainty structure of progressively censored lifetime data.

The normalization of the weighting scheme also clarifies its behavior in the absence of censoring. When complete observations are available, $R_{i}=0$ for all i=1,…,m. Consequently, *m = n* and $w_{i}=\frac{1}{n}$, so that the adjusted entropy statistic at monitoring epoch *t* becomes

$${\hat{H}}_{t}^{adj}=-\frac{1}{n}\sum_{i=1}^{n}logf\left(x_{i};{\hat{\alpha }}_{t},{\hat{\beta }}_{t},{\hat{\lambda }}_{t}\right).$$

Thus, under complete observations, all observations contribute equally to the entropy statistic. Under Progressive Type-II censoring, in contrast, the weights $w_{i}=\frac{1+R_{i}}{n}$ provide a normalized representation of the corresponding censoring stages. Hence, the proposed weighting scheme naturally connects the censoring-adjusted entropy statistic with its complete-data counterpart.

## 3. The Proposed Entropy EWMA Monitoring Scheme for Information Degradation

In this section, a novel monitoring framework is proposed to detect changes in the information structure of progressively censored lifetime observations. Unlike conventional monitoring procedures that mainly focus on shifts in quantiles, process location, or central tendency measures, the proposed methodology aims to identify latent degradation patterns through variations in uncertainty and information content.

The proposed monitoring framework is developed under the Exponentiated Weibull distribution and integrates a censoring-adjusted entropy measure into an adaptive exponentially weighted moving average (Entropy-EWMA) structure. This approach is motivated by the idea that process deterioration does not arise solely through changes in central tendency, but also through distortions in the information structure of lifetime observations.

For ease of reference, the main notation used in the proposed monitoring framework is summarized in Table A1 of Appendix A.

### 3.1. Entropy Degradation Mechanism

To formally represent process deterioration from an information-theoretic perspective, an entropy degradation mechanism is introduced. Unlike conventional monitoring frameworks where degradation is modeled through shifts in location or scale parameters, the proposed approach assumes that process deterioration appears through changes in uncertainty and information content. Let

$$H_{0}=-E[logf\left(X\right)]$$

denote the baseline entropy corresponding to the in-control process. To represent information degradation, the entropy structure is perturbed as

$$H_{1}=H_{0}+\Delta_{H},$$

where $\Delta_{H}$ denotes the true entropy-degradation magnitude imposed in the simulation. This notation is deliberately distinguished from the data-driven degradation estimate $\delta_{t}$ used subsequently in the monitoring procedure. Under $\Delta_{H}=0$, the process remains in-control, whereas larger values of $\Delta_{H}$ indicate increasing distortion in the underlying information structure. The degradation mechanism defines deviations from the baseline information state through the adjusted entropy estimator, which is subsequently standardized and transformed into the monitoring statistic in Section 3.2.

In the simulation study, information degradation is introduced by perturbing the entropy statistic after parameter estimation while preserving the underlying lifetime generation mechanism.

### 3.2. Construction of the Entropy–EWMA Monitoring Statistic

The proposed monitoring procedure consists of two distinct stages: an in-control calibration stage (Phase I) and a sequential monitoring stage (Phase II). In Phase I, the reference mean and standard deviation of the adjusted entropy statistic are estimated under in-control conditions. In Phase II, a new progressively Type-II censored sample is observed at each monitoring epoch t=1,2,… and its departure from the established in-control information structure is sequentially evaluated. This distinction is important because the Phase I quantities are estimated once and subsequently used as reference values throughout Phase II monitoring.

Let ${\hat{H}}_{t}^{adj}$ denote the adjusted entropy estimate obtained from the progressively Type-II censored sample observed at monitoring epoch *t*, as defined in Section 2.3. The first step of Phase II monitoring is to express the current entropy estimate relative to its in-control reference distribution. For this purpose, the standardized entropy deviation is defined as

(4) $$Z_{t}=\frac{{\hat{H}}_{t}^{adj}-{\hat{\mu }}_{H}}{{\hat{\sigma }}_{H}}$$

where ${\hat{\mu }}_{H}$ and ${\hat{\sigma }}_{H}$ are the Phase I estimates of the in-control mean and standard deviation of the adjusted entropy statistic, respectively. The standardization converts the adjusted entropy into a dimensionless deviation from the in-control information baseline. Consequently, $Z_{t}=0$ corresponds to the estimated in-control entropy level, while increasingly positive or negative values indicate progressively larger departures from that reference level. Standardization also prevents the subsequent monitoring statistic from depending directly on the numerical scale of the entropy measure and facilitates comparison across monitoring epochs and censoring settings.

Rather than determining the amount of smoothing from the current observation alone, the proposed procedure first constructs a smoothed estimate of the persistent entropy deviation. Specifically,

(5) $${\hat{\delta }}_{t}^{*}=\Psi Z_{t}+(1-\Psi ){\hat{\delta }}_{t-1}^{*}$$

with

$${\hat{\delta }}_{0}^{*}=0,$$

where Ψ∈(0,1] is the auxiliary shift-estimation smoothing parameter. The role of Ψ is to control the relative contribution of the current standardized entropy deviation and the accumulated historical information. Smaller values of Ψ assign greater weight to past observations and produce a smoother estimate that is more resistant to isolated fluctuations, whereas larger values place greater emphasis on the most recent entropy deviation and therefore produce a faster response to abrupt changes. Thus, Ψ controls the memory of the degradation estimator rather than serving as the adaptive EWMA smoothing coefficient of the Entropy–EWMA plotting statistic.

Because ${\hat{\delta }}_{0}^{*}=0$, the weights accumulated in Equation (5) do not sum to one during the initial monitoring epochs. To remove this initialization effect, the smoothed deviation is normalized by the cumulative EWMA weight:

(6) $${\hat{\delta }}_{t}^{**}=\frac{{\hat{\delta }}_{t}^{*}}{1-{\left(1-\Psi \right)}^{t}}$$

The denominator approaches one as *t* increases, so this correction primarily affects the beginning of the monitoring sequence. Under an in-control process, the standardized entropy deviations are centered around zero and hence ${\hat{\delta }}_{t}^{**}$ is expected to fluctuate around zero. Persistent departures from the in-control entropy structure cause the magnitude of ${\hat{\delta }}_{t}^{**}$ to increase.

Since deterioration may manifest itself through either an increase or a decrease in the entropy relative to its in-control reference level, the magnitude of the estimated deviation is used to determine the adaptive response:

(7) $$\delta_{t}=\left|{\hat{\delta }}_{t}^{**}\right|.$$

Thus, $\delta_{t}$ is not an additional observed quantity or model parameter; rather, it is a data-driven estimate of the magnitude of the persistent standardized entropy departure accumulated up to monitoring epoch *t*. Small values of $\delta_{t}$ indicate that the information structure remains close to its in-control reference, whereas larger values indicate stronger evidence of a sustained entropy departure.

It is important to distinguish the roles of Ψ and the adaptive EWMA smoothing coefficient in the proposed monitoring procedure. The parameter Ψ is the auxiliary shift-estimation smoothing parameter and controls the relative contribution of the current standardized entropy deviation $Z_{t}$ and its historical information in estimating the persistent degradation magnitude. In contrast, the adaptive coefficient controls the smoothing of the final plotting statistics $W_{t}$. Thus, Ψ does not directly determine the smoothing of $W_{t}$; instead, it governs the evolution of the auxiliary degradation estimates, which subsequently determine the adaptive coefficient used in the monitoring statistic.

To preserve the strictly sequential structure of the monitoring procedure, the adaptive EWMA smoothing coefficient applied at monitoring epoch *t* is determined using the degradation information available at the preceding epoch. Accordingly,

$$\lambda_{t}=\lambda {(\delta }_{t-1})$$

where $\delta_{t-1}=\left|{\hat{\delta }}_{t-1}^{**}\right|$. This one-step sequential update prevents the current standardized entropy deviation $Z_{t}$ from being used simultaneously both to determine the adaptive smoothing coefficient and to update the plotting statistic. For initialization, $\delta_{0}=0$ is assumed, yielding $\lambda_{1}=\lambda \left(0\right)=0.05$.

Accordingly, the proposed Entropy–EWMA plotting statistic is recursively defined as

(8) $$W_{t}=\lambda {(\delta }_{t})Z_{t}+\left(1-\lambda \left(\delta_{t}\right)\right)W_{t-1},\;W_{0}=0.\;$$

The adaptive smoothing function is defined as

(9) $$\lambda (\delta_{t})=\left(\begin{array}{cc} 0.05, & 0\leq \delta_{t}<0.25\\ 0.10, & 0.25\leq \delta_{t}<0.75\\ 0.20, & 0.75\leq \delta_{t}<1.5\\ 0.40, & 1.5\leq \delta_{t}<2.5\\ 0.70, & 2.5\leq \delta_{t}<3.5\\ 1.00, & \delta_{t}\geq 3.5 \end{array}\right).$$

This sequential adaptive structure gives the monitoring statistic a long memory when the previously estimated entropy departure is small and progressively increases the weight assigned to the current standardized entropy statistic when stronger persistent degradation has already been identified. Consequently, small or moderate persistent departures are accumulated through stronger smoothing, whereas larger degradation estimates lead to a faster response of the monitoring statistic. The threshold values defining the intervals of $\delta_{t-1}$ and the corresponding values of $\lambda {(\delta }_{t-1})$ were determined through preliminary calibration experiments designed to provide a practical balance between in-control run-length stability and out-of-control detection sensitivity.

For a simple numerical illustration, consider three successive standardized entropy deviations $Z_{1}=0.20$, $Z_{2}=0.40$, and $Z_{3}=1.50$. For Ψ=0.10, application of the recursive and bias-corrected shift estimator gives $\delta_{1}=0.200$, $\delta_{2}=0.305$, and $\delta_{3}=0.746$. In particular, at t=3,

$${\hat{\delta }}_{3}^{**}=\frac{0.10\left(1.50\right)+0.09\left(0.40\right)+0.081(0.20)}{1-0.9^{3}}\approx 0.746$$

According to the adaptive rule, this value corresponds to $\lambda {(\delta }_{3})=0.10$. In contrast, for Ψ=0.50, the same sequence produces $\delta_{1}=0.200$, $\delta_{2}=0.333$, and $\delta_{3}=1.000$, with

$${\hat{\delta }}_{3}^{**}=\frac{0.50\left(1.50\right)+0.25\left(0.40\right)+0.125\left(0.20\right)}{1-0.5^{3}}=1$$

Under the sequential adaptive rule, these degradation estimates determine the smoothing coefficient used at the next monitoring epoch. Hence, for Ψ=0.10,

$$\delta_{3}=0.746\;\;\;\Rightarrow \;\;\lambda_{4}=\lambda \left(\delta_{3}\right)=0.10$$

whereas for Ψ=0.50,

$$\delta_{3}=1\;\;\;\Rightarrow \;\;\lambda_{4}=\lambda \left(\delta_{3}\right)=0.20.$$

Thus, although Ψ does not enter the $W_{t}$ recursion directly, changing Ψ alters the estimated degradation trajectory and may place the previous degradation estimate in a different interval of the adaptive rule, thereby changing the smoothing coefficient applied at the subsequent monitoring epoch. Across repeated samples, this mechanism affects both the distribution of the estimated degradation levels and the frequency with which the different adaptive smoothing coefficients are selected.

The auxiliary shift-estimation smoothing parameter Ψ and the adaptive EWMA smoothing coefficient therefore perform distinct functions. The parameter Ψ is used to obtain a smoothed estimate of the magnitude of the persistent entropy departure, whereas $\lambda \left(\delta_{t-1}\right)$ determines how rapidly the final plotting statistic responds at the current monitoring epoch based on the degradation information accumulated up to the preceding epoch. In the simulation study, several values of Ψ, namely 0.05, 0.10, 0.25, 0.50, and 0.80, are examined to assess the effect of this auxiliary smoothing choice on monitoring performance rather than imposing a single arbitrary value.

At each monitoring epoch *t*, the resulting statistic is compared with the predetermined control limit *h*. An out-of-control signal is generated whenever

(10) $$\left|W_{t}\right|>h.$$

The control limit *h* is determined through Monte Carlo calibration under the in-control model. For a given censoring scheme and monitoring configuration, candidate values of *h* are evaluated by repeatedly generating in-control progressively Type-II censored samples and computing the corresponding run lengths. The value of *h* that produces an estimated in-control Average Run Length closest to the prespecified target ${ARL}_{0}=370$ is selected as the control limit. This calibration is performed before evaluating out-of-control performance, and the resulting value of *h* is subsequently held fixed for the corresponding monitoring configuration. If $\left|W_{t}\right|\leq h$, no signal is generated and the procedure proceeds to monitoring epoch *t* + 1, where a new progressively Type-II censored sample is observed and the entire sequential update is repeated.

For clarity, the complete Phase II monitoring procedure can therefore be summarized as follows. At monitoring epoch *t*:

(i) obtain the current progressively Type-II censored sample;
(ii) estimate the Exponentiated Weibull parameters ${(\hat{\alpha }}_{t},{\hat{\beta }}_{t},{\hat{\lambda }}_{t})$ by maximum likelihood;
(iii) calculate the adjusted entropy estimate ${\hat{H}}_{t}^{adj}$;
(iv) standardize it relative to the Phase I reference distribution to obtain $Z_{t}$;
(v) determine the adaptive coefficient for the current epoch as $\lambda_{t}=\lambda {(\delta }_{t-1})$, with $\delta_{0}=0$;
(vi) update the Entropy–EWMA plotting statistic $W_{t}$;
(vii) update the auxiliary estimator ${\hat{\delta }}_{t}^{*}$, obtain its initialization-corrected value ${\hat{\delta }}_{t}^{**}$, and calculate $\delta =\left|{\hat{\delta }}_{t}^{**}\right|$, which is retained for determining $\lambda_{t+1}$;
(viii) compare $\left|W_{t}\right|$ with *h*. If the control limit is exceeded, an out-of-control signal is produced; otherwise, monitoring continues with the next sample.

The performance of the proposed Entropy–EWMA chart is evaluated using run-length characteristics, particularly the Average Run Length (ARL) and Standard Deviation of Run Length (SDRL). Monte Carlo simulations are used to investigate both in-control and out-of-control performance under different censoring schemes, observed sample sizes, auxiliary smoothing settings, and entropy-degradation scenarios.

## 4. Performance Evaluation

The statistical performance of the proposed Entropy–EWMA monitoring scheme is evaluated through Monte Carlo simulation using run-length characteristics. Let the run length (RL) denote the number of monitoring epochs required for the chart to produce its first out-of-control signal. Formally,

$$RL=inf\{t\geq 1:\left|W_{t}\right|>h\}$$

where $W_{t}$ is the Entropy–EWMA plotting statistic defined in Section 3.2 and *h* denotes the calibrated control limit. Thus, RL directly measures the number of monitoring epochs required for the proposed procedure to signal a departure from the in-control information structure.

The interpretation of RL depends on the state of the process. Under in-control conditions, a large RL is desirable because it indicates a low frequency of false alarms. Accordingly, the in-control Average Run Length, denoted by ${ARL}_{0}$, is calibrated to approximately 370. Under out-of-control conditions, in contrast, smaller run lengths are desirable because they indicate faster detection of information degradation. Therefore, the detection performance of the proposed monitoring scheme is evaluated under approximately comparable ${ARL}_{0}$ conditions, with smaller out-of-control ARL values indicating better detection performance.

For each experimental configuration, 5000 Monte Carlo replications are performed. If ${RL}_{r}$ denotes the run length obtained from the r*th* replication, r=1,…,5000, the mean and standard deviation of the run length are calculated as

$$MeanRL=\frac{1}{5000}\sum_{r=1}^{5000}{RL}_{r}$$

and

$$SDRL={\left[\frac{1}{4999}\sum_{r=1}^{5000}{\left({RL}_{r}-MeanRL\right)}^{2}\right]}^{1/2}$$

MeanRL represents the average number of monitoring epochs required to generate a signal, whereas SDRL describes the variability of the signaling time across simulation replications. Under out-of-control conditions, a smaller MeanRL indicates faster detection, while a smaller SDRL, particularly when MeanRL values are comparable, indicates more consistent detection performance.

In addition to MeanRL and SDRL, selected quantiles of the run-length distribution are reported to provide a more complete description of monitoring performance. Let

$$QRL\left(p\right)=\inf\left\{r:P\left(RL\leq r\right)\geq p\right\},\;\;\;0<p<1$$

denote the p*th* quantile of the run-length distribution. In Table 1, Table 2, Table 3, Table 4 and Table 5, $QRL\left(0.05\right)$, $QRL\left(0.25\right)$, $QRL\left(0.50\right)$, and $QRL\left(0.75\right)$ represent the 5*th*, 25*th*, 50*th*, and 75*th* percentiles of the simulated run lengths, respectively. Under in-control conditions, larger lower-tail quantiles indicate greater resistance to early false alarms. Under out-of-control conditions, smaller run-length quantiles indicate earlier detection of process degradation. Hence, MeanRL, SDRL, and the run-length quantiles jointly characterize the average detection speed, variability, and distribution of signaling times.

The simulations are conducted under an in-control Exponentiated Weibull model with baseline parameters

$$\alpha^{\left[0\right]}=1.5,\;\;\beta^{\left[0\right]}=1.2,\;\;\lambda^{\left[0\right]}=1.$$

The total number of units placed on test is fixed at *n* = 100, while three numbers of observed failures are considered:

m=20, 30, 40,

representing different levels of information retained under Progressive Type-II censoring. Two censoring structures, Uniform and Late, are examined. Under the Uniform censoring structure, removals are distributed approximately evenly across the observed failure times, whereas under the Late censoring structure, most removals occur toward the final stages of the life test. These censoring schemes are considered to assess the behavior of the proposed monitoring framework under different patterns of information removal. These censoring structures follow the Progressive Type-II censoring framework described in Section 2.2. For reproducibility, the exact Progressive Type-II censoring vectors used for each observed sample size are reported in Table 1.

Here, $a^{\left(k\right)}$ denotes that the value a is repeated k consecutive times. For each configuration, the censoring vector satisfies $\sum_{i=1}^{m}R_{i}=n-m$. The same censoring vectors corresponding to *n* = 100 and *m* = 30 were subsequently used in the real-data application.

The influence of the auxiliary shift-estimation smoothing parameter Ψ introduced in Section 3.2 is investigated using

Ψ=0.05, 0.10, 0.25, 0.50, 0.80.

For each combination of Ψ, *m*, and censoring scheme, the control limit *h* is calibrated through Monte Carlo simulation under in-control conditions so that the resulting ${ARL}_{0}$ is approximately 370. Once calibrated, the corresponding value of *h* is held fixed when evaluating the out-of-control scenarios for the same monitoring configuration.

To evaluate the ability of the proposed chart to detect changes in the information structure, three entropy degradation levels are considered:

$$\Delta_{H}=0,\;0.50,\;1.50.\;$$

The implementation of entropy degradation is defined explicitly as an additive perturbation to the adjusted entropy statistic. At monitoring epoch *t*, after the Exponentiated Weibull parameters have been estimated from the progressively censored sample and the adjusted entropy statistic ${\hat{H}}_{t}^{adj}$ has been calculated, the perturbed entropy statistic is defined as

$${\hat{H}}_{t}^{adj}={\hat{H}}_{t}^{adj}+\delta_{H}.$$

Here, $\delta_{H}=0$ represents the in-control information state, while $\delta_{H}=0.50$ and $\delta_{H}=1.50$ represent moderate and severe entropy degradation, respectively. The perturbation is introduced after parameter estimation so that the underlying lifetime-generation mechanism remains unchanged. In this way, the simulation specifically evaluates the ability of the proposed monitoring scheme to detect departures in the entropy structure without imposing a predetermined change on an individual Exponentiated Weibull parameter.

For each Monte Carlo replication, a progressively Type-II censored sample is first generated according to the specified censoring scheme. The Exponentiated Weibull parameters are then estimated by maximum likelihood, and the adjusted entropy statistic is calculated. For an out-of-control scenario, the specified entropy perturbation $\delta_{H}$ is subsequently applied. The resulting entropy statistic is standardized using the in-control reference mean and standard deviation established during Phase I. The preliminary degradation estimator and adaptive smoothing coefficient are then updated as described in Section 3.2, and the Entropy–EWMA plotting statistic $W_{t}$ is recursively calculated. This sequential procedure continues until $\left|W_{t}\right|>h$, at which point the corresponding monitoring epoch is recorded as the run length. The procedure is repeated 5000 times for each experimental configuration, and the resulting run-length characteristics are summarized in Table 1, Table 2, Table 3, Table 4 and Table 5.

In practical monitoring applications, the statistical performance of a control chart is generally evaluated using run length characteristics including Average Run Length (ARL), Standard Deviation of Run Length (SDRL), and selected quantiles of the run length (RL) distribution. Among these measures, ARL is considered the primary performance criterion in the present study. A monitoring scheme is considered effective if, for a fixed in-control ARL, the out-of-control ARLs decrease rapidly as the level of process degradation increases. Since the proposed Entropy-EWMA framework represents a new information-theoretic monitoring approach developed for progressively Type-II censored lifetime data, its statistical performance is investigated under various parameter configurations and censoring scenarios.

The performance evaluation is conducted using Monte Carlo simulation with 5000 replications for each experimental setting. For each replication, progressively Type-II censored samples are generated from the Exponentiated Weibull distribution and the corresponding run length values are recorded.

Throughout the simulation study, the in-control process is calibrated to achieve approximately $ARL_{0}=370$ which is a commonly accepted benchmark in statistical process monitoring studies. The initial parameter values of the Exponentiated Weibull distribution are selected as

$$\alpha^{\left[0\right]}=1.5,\;\beta^{\left[0\right]}=1.2,\;\lambda^{\left[0\right]}=1$$

To investigate the effect of information availability under censoring, the total sample size is fixed as n=100 while different observed sample sizes are considered: m=20, 30, 40.

To evaluate the ability of the proposed chart to detect changes in the information structure, several degradation levels are considered. The imposed entropy degradation magnitude $\Delta_{H}$ is varied as

$$\Delta_{H}=0,\;0.50,\;1.50$$

Here, $\Delta_{H}=0$ represents the in-control state, whereas larger values correspond to progressively stronger distortions in the information structure.

The influence of the auxiliary shift-estimation smoothing parameter Ψ on monitoring performance is also examined. The following values are considered:

Ψ=0.05, 0.10, 0.25, 0.50, 0.80

In addition, two Progressive Type-II censoring structures are considered to evaluate the robustness of the proposed monitoring framework under different information removal mechanisms. Specifically, Uniform and Late censoring schemes are adopted. Under the Uniform censoring structure, censored observations are removed approximately evenly throughout the experiment, whereas under the Late censoring structure, most removals occur at the final stages of the life test. Early censoring structures are not included in order to avoid excessive information loss at the initial stages of monitoring. Furthermore, to investigate the effect of information availability under censoring, the total sample size is fixed at *n* = 100, while three observed sample sizes are considered: *m* = 20, 30, 40 representing different levels of available information under progressively censored observations.

For each simulated sample, the proposed monitoring framework is implemented according to the following procedure. First, a progressively Type-II censored sample is generated from the Exponentiated Weibull distribution.

For each combination of Ψ, *m*, and censoring scheme, the control limit *h* is calibrated through Monte Carlo simulation under in-control conditions so that the resulting $ARL_{0}$ is approximately 370. The calibration is performed over a fine grid of candidate control-limit values in the interval [0.10, 6.00], with a grid resolution of 0.0025. For each configuration, 5000 Monte Carlo replications are used to select the value of h producing the simulated in-control ARL closest to the target $ARL_{0}=370$. The selected control limit is subsequently evaluated using an independent set of 5000 Monte Carlo replications. A validation tolerance of ±30 around the nominal target is adopted; if the independently estimated ARL falls outside this range, an additional local search over neighboring control-limit values is performed. Because the calibration criterion is based on Monte Carlo estimates and *h* is selected from a discrete numerical grid, exact equality to 370 is neither expected nor imposed. The remaining deviations from the nominal target reflect Monte Carlo variability together with the finite resolution of the control-limit search. Importantly, the maximum simulated run length was increased to 5000, and no run-length censoring occurred in the reported simulations.

**Figure 1:**
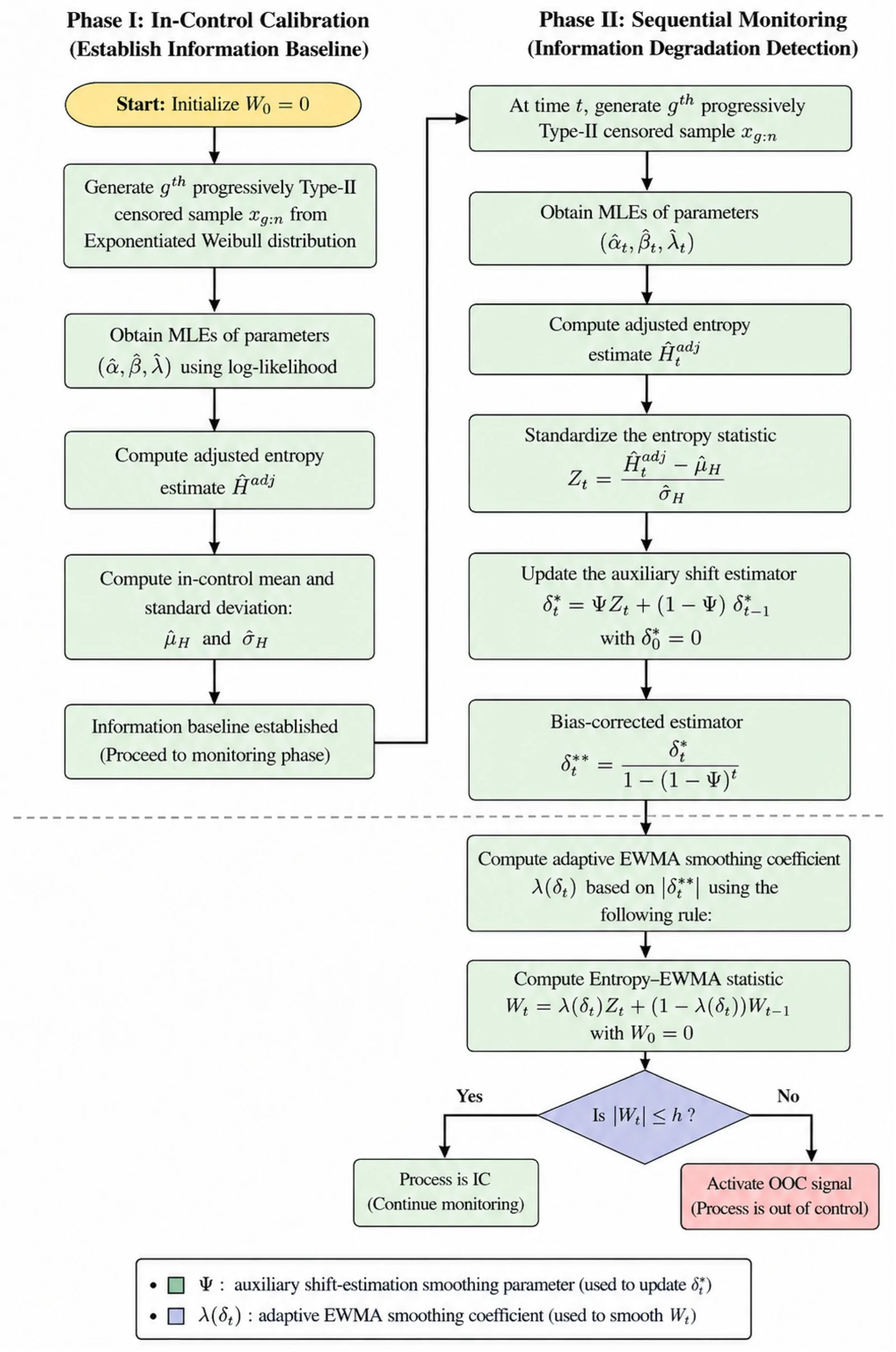
Flowchart for the monitoring scheme.

Figure 1 summarizes the complete workflow of the proposed Entropy–EWMA monitoring framework. Phase I establishes the in-control reference entropy distribution through maximum likelihood parameter estimation and adjusted entropy calculation. Phase II performs sequential monitoring by transforming each newly observed progressively Type-II censored sample into a standardized entropy statistic, estimating the magnitude of the persistent information departure, determining the adaptive smoothing coefficient, and recursively updating the Entropy–EWMA statistic until an out-of-control signal is generated. Thus, the proposed framework continuously compares the current information structure with the established in-control baseline while adaptively adjusting its memory according to the estimated degradation level. In this way, the procedure is designed to detect sustained changes in the overall uncertainty structure of the lifetime process rather than relying exclusively on changes in a particular distributional parameter, the process mean, or a selected quantile.

Table 2 shows that the proposed Entropy–EWMA scheme maintains satisfactory in-control performance under both censoring structures, with ARL values remaining close to the target ARL0 = 370. As the entropy degradation level increases, ARL decreases sharply and consistently, indicating rapid detection of moderate and severe information deterioration.

**Figure 2:**
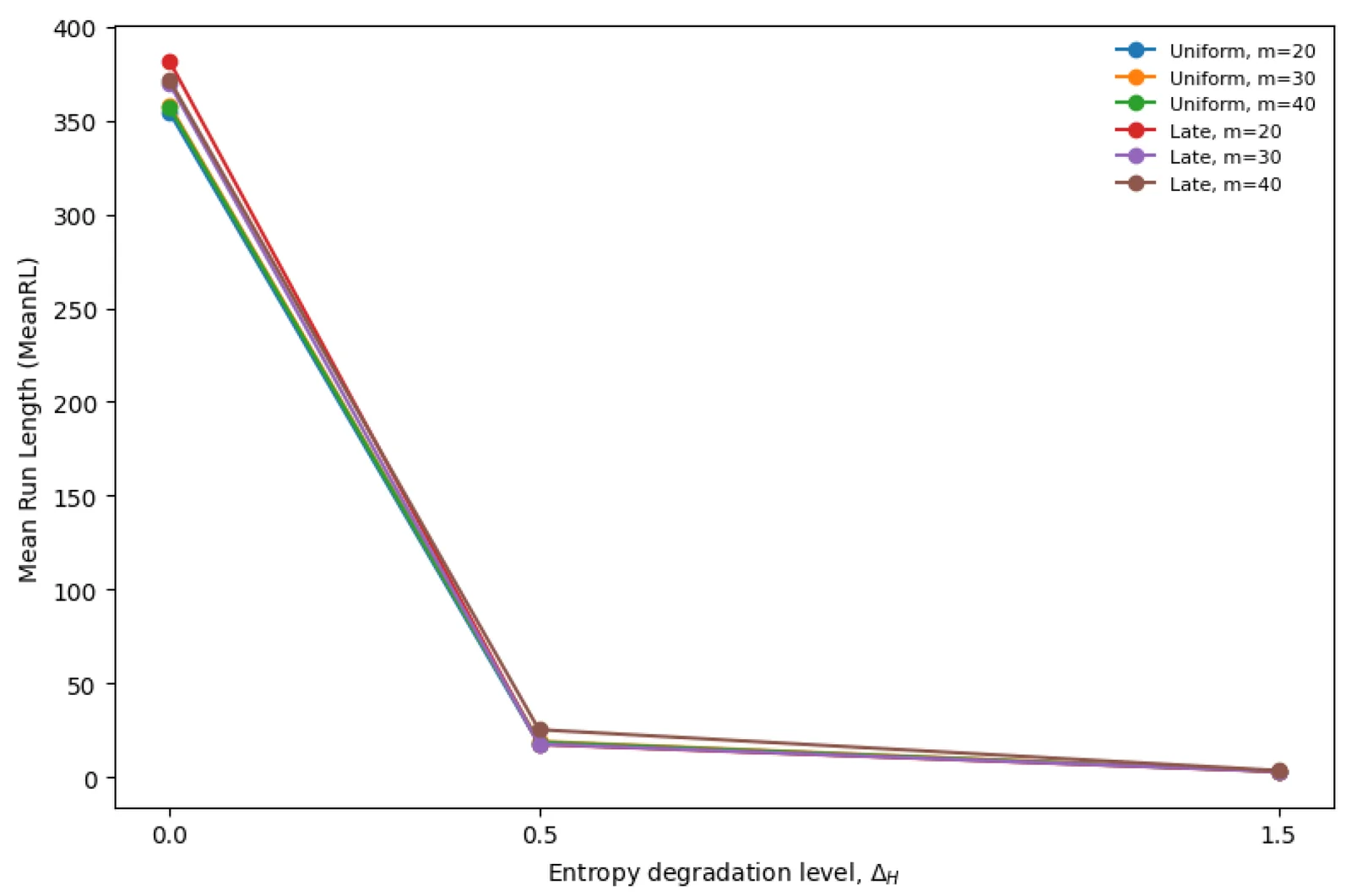
Mean run length (ARL) under increasing entropy degradation levels for Uniform and Late Progressive Type-II censoring structures with Ψ=0.05, n=100.

Figure 2 clearly illustrates the rapid decrease in ARL as the entropy degradation magnitude increases. While the in-control ARL values remain close to the nominal target, even a moderate degradation $\Delta_{H}=0.50$ produces a substantial reduction in detection time, and severe degradation $\Delta_{H}=1.50$ is detected within only a few monitoring epochs. The similar patterns observed under Uniform and Late censoring further indicate that the proposed monitoring scheme maintains consistent detection behavior across the considered censoring structures.

Table 3 indicates that the monitoring performance remains broadly consistent across the different observed sample sizes when Ψ=0.10. For moderate degradation $\Delta_{H}=0.50$, ARL values are generally concentrated around 20–23, although the Late censoring structure with *m =* 40 exhibits a somewhat slower response (ARL = 29.83). Under severe degradation $\Delta_{H}=1.50$, this difference largely disappears, with ARL remaining below 4 for all configurations.

**Figure 3:**
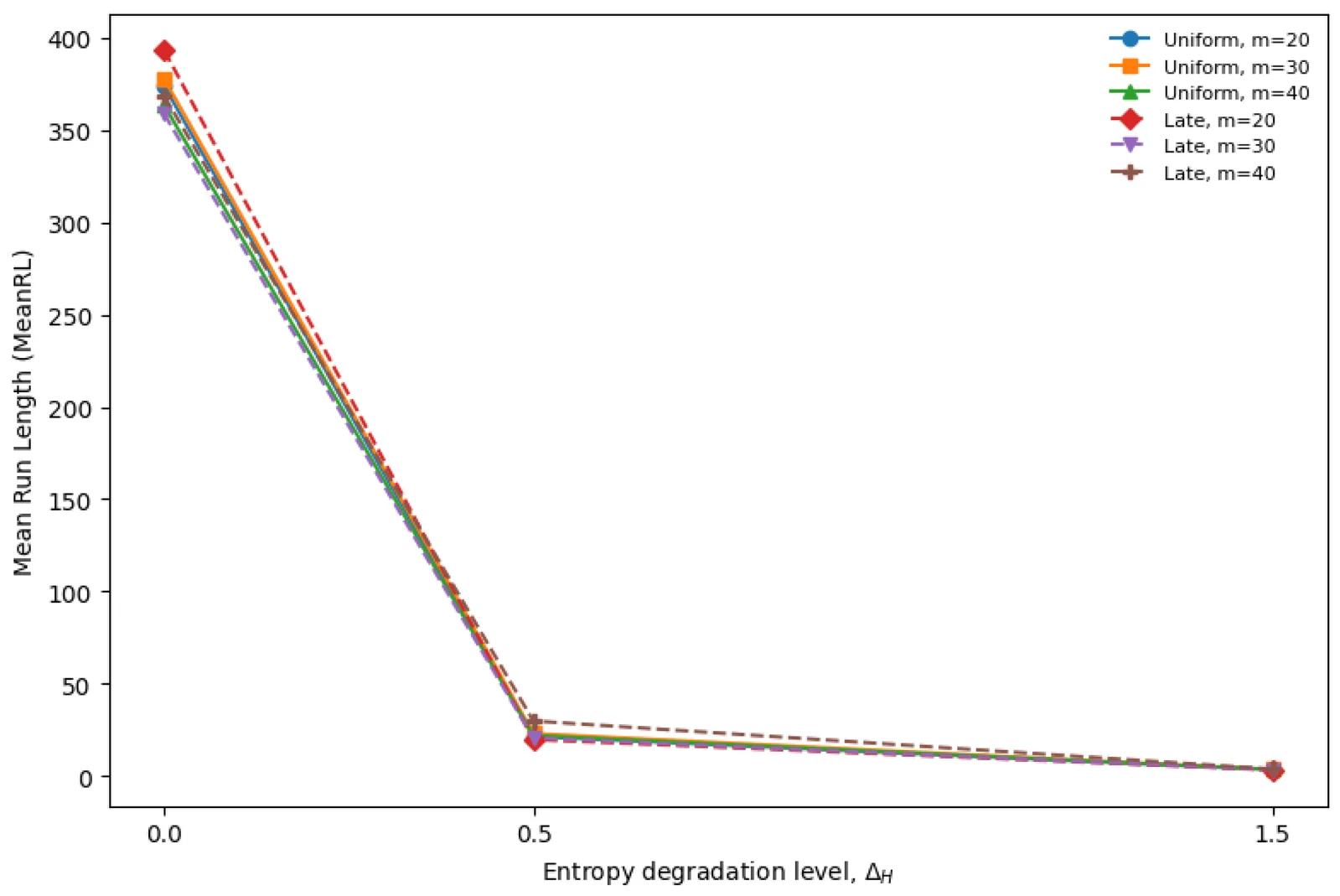
Mean run length (ARL) under increasing entropy degradation levels for Uniform and Late Progressive Type-II censoring structures with Ψ=0.10, n=100.

Figure 3 shows a strong convergence of the run-length profiles as the degradation magnitude increases. Differences among censoring configurations are most visible in the in-control state, become substantially smaller at $\Delta_{H}=0.50$, and are almost negligible at $\Delta_{H}\;=1.50$. This suggests that severe information deterioration dominates the effects of both the censoring structure and the observed sample size on detection speed.

Table 4 demonstrates particularly stable in-control calibration for Ψ=0.25, with ARL values ranging from 359.05 to 374.48 across all censoring configurations. At the moderate degradation level, Uniform censoring yields relatively similar detection performance across *m* = 20, 30, and 40, whereas Late censoring with *m* = 40 produces a noticeably larger ARL. Nevertheless, severe degradation is detected rapidly in every configuration, with ARL remaining close to four.

**Figure 4:**
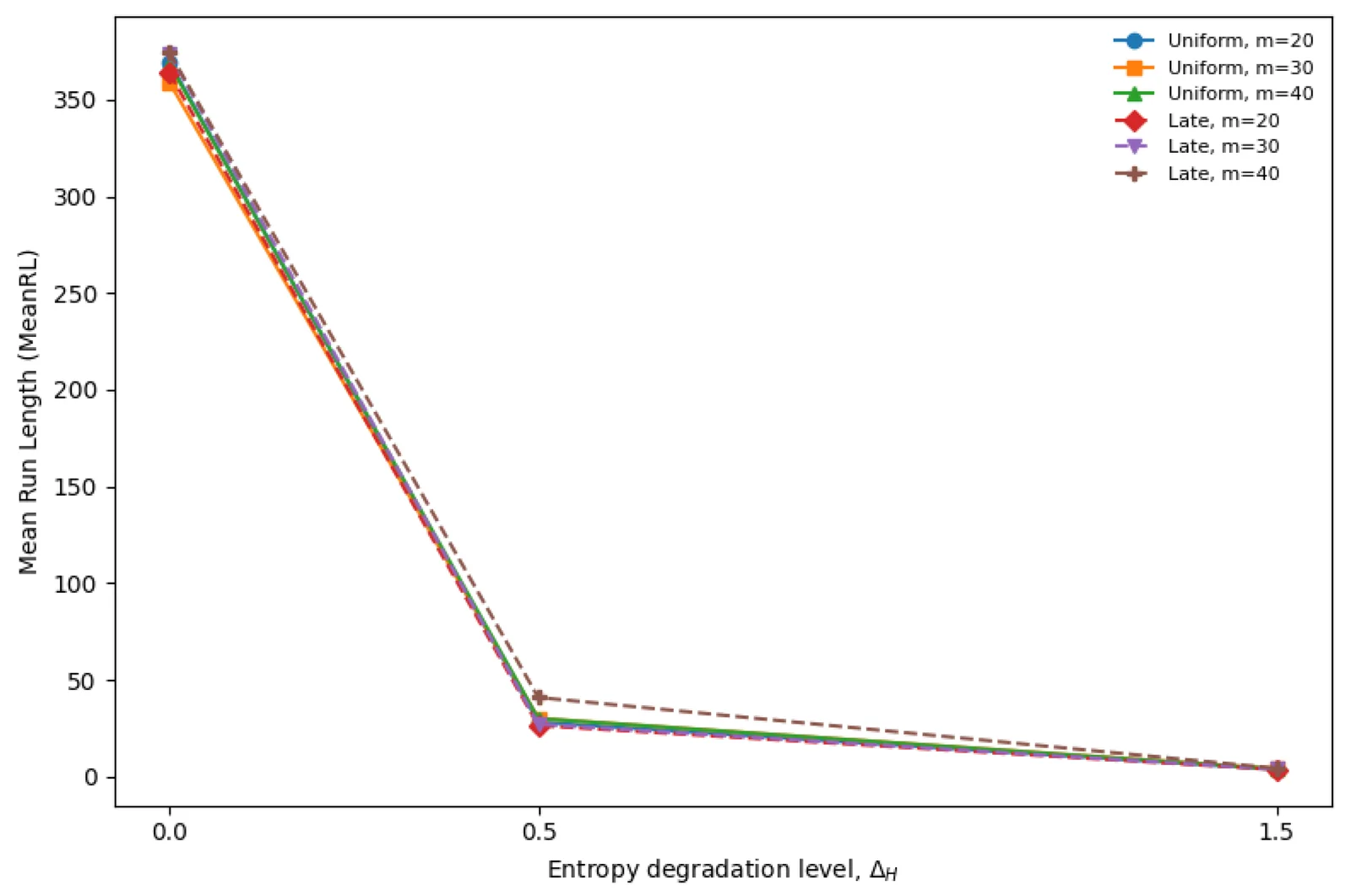
Mean run length (ARL) under increasing entropy degradation levels for Uniform and Late Progressive Type-II censoring structures with Ψ=0.25, n=100.

Figure 4 highlights that the effect of the censoring configuration is most pronounced at the intermediate degradation level. In particular, the Late censoring scheme with *m* = 40 shows a higher ARL than the remaining configurations at $\Delta_{H}=0.50$, while the curves become closely grouped again under severe degradation. This pattern suggests that the censoring design has its greatest influence on detection efficiency when the information deterioration is moderate rather than pronounced.

Table 5 shows that, for Ψ=0.50, the in-control run-length distributions are shifted away from very early signaling, with $Q_{RL}$ (0.05) ranging from 15 to 18. Once degradation is introduced, however, the lower and median run-length quantiles decrease markedly. For severe degradation, the median run length is only 4 across all configurations, indicating highly concentrated and rapid detection despite differences in censoring design.

**Figure 5:**
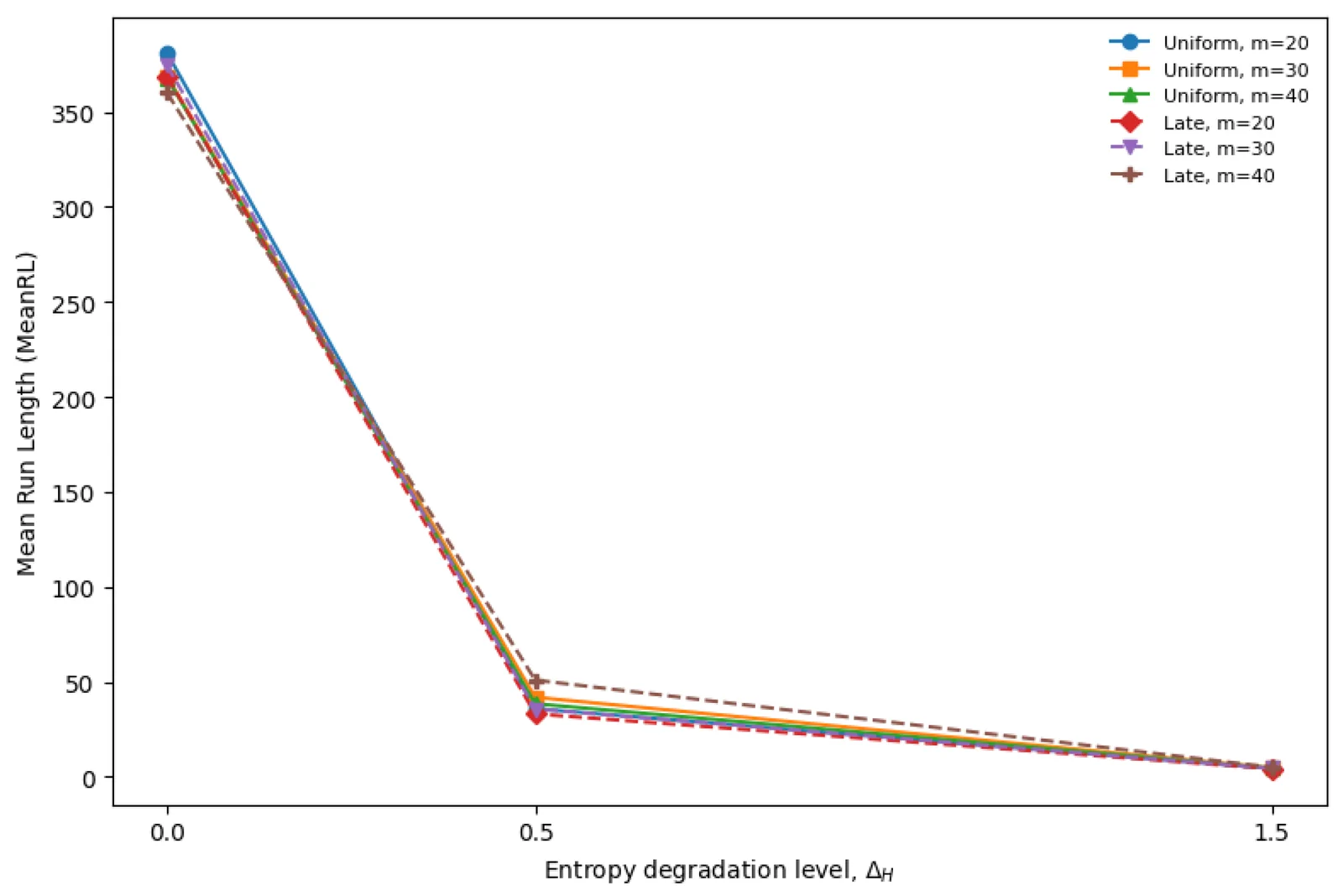
Mean run length (ARL) under increasing entropy degradation levels for Uniform and Late Progressive Type-II censoring structures with Ψ=0.50,  n=100.

Figure 5 reveals a relatively compact response pattern among the three Uniform censoring configurations, particularly under out-of-control conditions. In contrast, Late censoring with *m* = 40 retains a somewhat higher ARL at the moderate degradation level. Despite this separation, all profiles collapse to very low run lengths at $\Delta_{H}=1.50$, showing that the influence of the censoring configuration becomes limited when the entropy departure is sufficiently large.

Table 6 shows that the relatively large auxiliary smoothing value Ψ=0.80 preserves well-calibrated in-control behavior, with ARL values between 361.25 and 371.47. However, detection of moderate entropy degradation is comparatively slower, with ARL values ranging from 47.89 to 68.74. In contrast, severe degradation remains readily detectable, producing ARL values of approximately 5–6 across all configurations.

**Figure 6:**
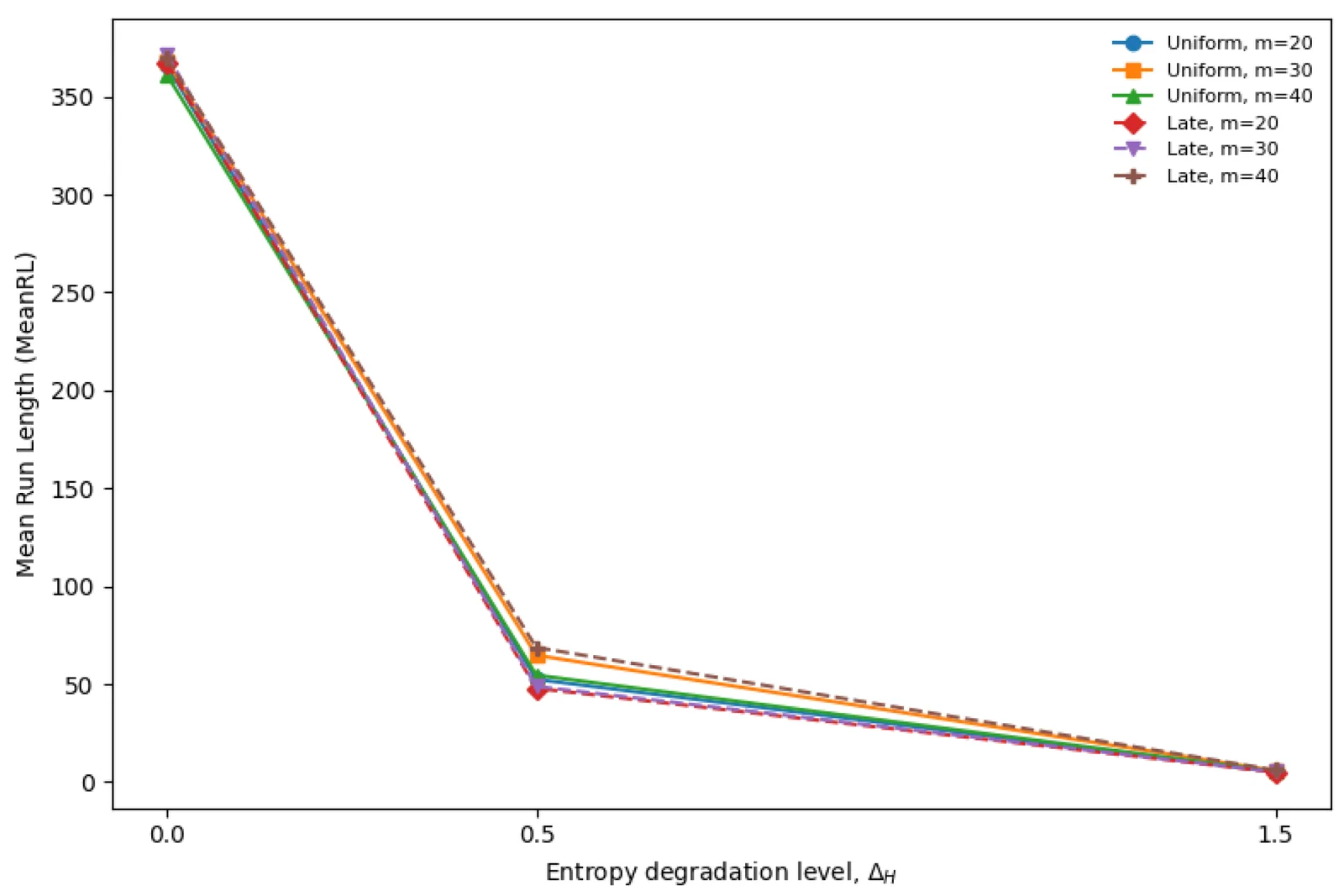
Mean run length (ARL) under increasing entropy degradation levels for Uniform and Late Progressive Type-II censoring structures with Ψ=0.80,  n=100.

Figure 6 exhibits a more gradual reduction in ARL from the in-control state to the moderate degradation level than that observed for smaller values of Ψ. This behavior reflects the reduced sensitivity of the monitoring mechanism to moderate information departures when the auxiliary estimator places greater emphasis on recent observations. Nevertheless, the profiles again approach one another under severe degradation, indicating that sufficiently large entropy departures are detected rapidly even at Ψ=0.80.

### 4.1. Simulation Results and Main Findings

Table 2, Table 3, Table 4, Table 5 and Table 6 summarize the run length performance of the proposed Entropy–EWMA monitoring framework under different values of the auxiliary shift-estimation smoothing parameter Ψ, entropy degradation levels $\delta_{H}$, observed sample sizes *m*, and Progressive Type-II censoring structures. Overall, the simulation results demonstrate that the proposed monitoring framework provides stable monitoring behavior while maintaining effective degradation detection capability under progressively censored lifetime observations.

Across all experimental settings, the proposed chart preserves acceptable in-control performance after control-limit calibration. For most scenarios with $\delta_{H}=0$, the ARL values remain reasonably close to the target ARL level, indicating that the monitoring procedure maintains satisfactory false alarm control under both Uniform and Late censoring structures.

A consistent trend observed throughout Table 2, Table 3, Table 4, Table 5 and Table 6 is that the out-of-control run length decreases as the entropy degradation level increases. Under both censoring schemes, larger entropy perturbations generate substantially earlier signals. For example, under Uniform censoring with *m* = 30, ARL decreases from 380.40 Ψ=0.05 to 160.47 for $\delta_{H}=0.50$ and further to 11.89 for $\delta_{H}=1.50$. Similar reductions are observed across all smoothing configurations, confirming that the proposed entropy-based monitoring statistic is sensitive to information deterioration. The auxiliary shift-estimation smoothing parameter Ψ has a clear impact on monitoring performance. Smaller and moderate values of Ψ generally provide the most balanced monitoring behavior by preserving stable in-control performance while maintaining relatively rapid out-of-control detection. In particular, Ψ=0.10 and Ψ=0.25 frequently yield favorable trade-offs between false alarm resistance and detection efficiency across different censoring scenarios.

As Ψ increases beyond moderate values Ψ=0.50 and Ψ=0.80, the chart becomes progressively more conservative. Although the in-control behavior remains stable after calibration, moderate entropy degradation requires longer run lengths before detection. For example, under Uniform censoring with *m* = 30 and $\delta_{H}=0.50$, ARL increases from 149.18 Ψ=0.10 to 316.54 Ψ=0.80, indicating delayed detection under moderate information shifts. Nevertheless, severe degradation $\delta_{H}=1.50$ continues to produce substantially reduced run lengths, showing that the monitoring statistic remains effective for strong deterioration scenarios.

The observed sample size m also influences monitoring performance. In general, larger observed sample sizes tend to stabilize the monitoring process and improve the consistency of detection under moderate degradation conditions. However, the improvement is not strictly monotonic across all censoring structures because the amount of retained information also depends on the censoring pattern. Regarding censoring structures, Uniform censoring generally provides slightly faster detection under moderate degradation levels, whereas Late censoring tends to preserve monitoring stability and produces relatively more conservative signaling behavior. Since Late censoring retains more observations until the later stages of the experiment, it preserves the information structure for longer periods and therefore delays signal generation in several scenarios.

In addition to ARL and SDRL, the reported run length quantiles provide further insight into monitoring behavior. Lower quantiles characterize early false alarm tendencies, whereas upper quantiles describe the variability of detection performance. Across all smoothing settings, these quantiles systematically decrease as entropy degradation increases, supporting the robustness of the proposed monitoring framework. Overall, the simulation results indicate that the proposed Entropy–EWMA monitoring scheme successfully adapts to different information degradation levels and Progressive Type-II censoring structures while maintaining a practical balance between monitoring stability and detection sensitivity. Among the investigated configurations, moderate smoothing values Ψ=0.10−0.25 provide the most satisfactory overall monitoring performance.

### 4.2. Comparative Performance Analysis with Classical EWMA

To further evaluate the practical effectiveness of the proposed monitoring framework, an additional comparative analysis was conducted against the classical EWMA chart. Since the proposed method is specifically designed to monitor entropy degradation rather than conventional location shifts, the comparison was performed under entropy-structure degradation scenarios while maintaining comparable in-control performance for both monitoring procedures. For the classical EWMA benchmark, the standardized monitoring statistics were generated by resampling from the empirical in-control reference distribution obtained from progressively Type-II censored Phase I samples. The control limits of the classical EWMA chart were calibrated separately for each censoring scheme to achieve an in-control ARL of approximately 370, thereby ensuring a comparable false-alarm basis for the comparison. The proposed Entropy–EWMA results correspond to the Ψ=0.25 and *m* = 30 configuration reported in Table 3.

Table 7 demonstrates a clear difference between the two monitoring approaches under entropy-structure degradation. Under the in-control condition $\Delta_{H}=0$, both charts exhibit ARL values close to the nominal target of 370, confirming that the comparison is conducted under comparable false-alarm conditions. When entropy degradation is introduced, the classical EWMA remains close to its in-control run-length level, indicating limited sensitivity to changes that primarily affect the information structure rather than the process location. In contrast, the proposed Entropy–EWMA chart responds strongly to increasing entropy degradation, with ARL decreasing to 30.13 and 4.08 under Uniform censoring and to 27.11 and 3.87 under Late censoring for $\Delta_{H}=0.5$ and 1.5, respectively. These results demonstrate the specific advantage of entropy-based monitoring for detecting information-structure deterioration that may remain largely undetected by conventional location-based monitoring.

## 5. Real Data Applications

### 5.1. Application to Wind Turbine SCADA Data

To demonstrate the practical applicability of the proposed Entropy–EWMA monitoring framework under progressively censored observations, a real industrial dataset obtained from a wind turbine supervisory control and data acquisition (SCADA) system was analyzed. The dataset was obtained from the publicly available Wind Turbine SCADA dataset [29] and consists of operational measurements collected from a wind turbine located in Turkey during the period from 1 January to 13 December 2018 at 10-min intervals. Among the available variables, wind speed (m/s) was selected for analysis since wind speed directly affects turbine performance and reflects variations in the underlying information structure of the process.

After preprocessing, observations containing missing, non-finite, or non-positive values were removed, resulting in a final dataset consisting of 50,520 valid wind speed measurements. Since the proposed methodology is developed for progressively Type-II censored lifetime monitoring, the original SCADA observations were transformed into progressively censored samples before constructing the monitoring statistics.

For the monitoring application, the total sample size was fixed at *n* = 100 and the observed sample size was selected as *m* = 30. Two Progressive Type-II censoring structures were considered in order to evaluate the behavior of the monitoring framework under different information removal mechanisms. The first structure corresponds to Uniform censoring, where censored observations are removed approximately evenly throughout the experiment. The second structure corresponds to Late censoring, where most observations remain available until the final stages and removals occur predominantly at the end of the monitoring period. These two censoring schemes were selected to represent balanced and information-preserving censoring scenarios.

For each monitoring step, a random sample of size *n* = 100 was generated from the SCADA observations and transformed into a progressively Type-II censored sample according to the selected censoring structure. For the proposed monitoring procedure, the adjusted entropy statistic was calculated from the censored sample and standardized relative to the Phase I reference distribution. The resulting standardized entropy deviation was then processed through the auxiliary shift-estimation mechanism and the adaptive smoothing rule described in Section 3.2 to obtain the Entropy–EWMA plotting statistic $W_{t}$. For comparison, a classical EWMA chart based on the standardized sample mean was also considered. Thus, both monitoring procedures were constructed relative to their corresponding in-control reference distributions.

The monitoring design followed a Phase I–Phase II structure. The first 30 monitoring samples were assumed to represent the in-control (IC) process and were used to establish baseline process behavior. The remaining 20 monitoring samples were treated as out-of-control (OOC) observations and represented process changes after the transition point $t_{0}=30$. Since the direction of entropy variation in real industrial data is not known a priori, a two-sided monitoring strategy was adopted, consistent with the simulation study, and an out-of-control signal was generated whenever $\left|W_{t}\right|>h$.

Before constructing the monitoring charts, descriptive statistics of the Wind Turbine SCADA dataset were examined to better understand the distributional characteristics of the wind speed measurements. The dataset was preprocessed by removing missing, non-finite, and non-positive observations, resulting in 50,520 valid measurements. Table 8 summarizes the main descriptive characteristics of the wind speed data, including location, variability, and shape measures. These statistics provide an initial characterization of the variability and distributional structure of the wind speed data prior to the model-fitting and monitoring analyses.

The Wind Turbine SCADA dataset consists of 50,520 valid wind speed observations collected after preprocessing and data cleaning. The average wind speed was estimated as 7.559 m/s with a standard deviation of 4.226 m/s, indicating moderate variability in turbine operating conditions. The coefficient of variation (0.559) suggests a moderate relative dispersion around the mean. The positive skewness value indicates a mildly right-skewed distribution. Overall, the observed variability and distributional characteristics provide a meaningful basis for the subsequent model-fitting and monitoring analyses.

To assess the adequacy of the Exponentiated Weibull distribution as the baseline model more comprehensively, its fit was compared with two alternative lifetime distributions, namely the Weibull and Generalized Gamma distributions. The comparison was based on the log-likelihood, Akaike Information Criterion (AIC), Bayesian Information Criterion (BIC), Kolmogorov–Smirnov (KS), Cramér–von Mises (CvM), and Anderson–Darling (AD) statistics. The results are presented in Table 9.

Table 9 shows that the formal goodness-of-fit statistics indicate detectable departures from all fitted models, particularly given the very large sample size. In particular, the CvM and AD statistics for the Exponentiated Weibull model should not be interpreted as indicating an exact distributional fit. Nevertheless, the comparative results consistently favor the Exponentiated Weibull distribution among the models considered. It provides the highest log-likelihood and the lowest AIC and BIC values, while also yielding the smallest KS, CvM, and AD statistics. The Generalized Gamma distribution provides a relatively close fit, whereas the Weibull distribution performs less favorably across the reported criteria. Therefore, the Exponentiated Weibull distribution was retained as the baseline model for the subsequent monitoring analysis based on its superior relative fit rather than on a claim of exact distributional adequacy.

Following the goodness-of-fit evaluation, the parameter estimates of the Exponentiated Weibull distribution were obtained using the maximum likelihood estimation procedure. In addition, approximate standard errors and 95% confidence intervals were calculated using the inverse Hessian matrix to assess estimation uncertainty. The estimation results are summarized in Table 10.

The maximum likelihood estimates indicate stable parameter estimation for the Exponentiated Weibull model fitted to the Wind Turbine SCADA data. The relatively small standard errors and narrow confidence intervals suggest acceptable estimation precision despite the large variability present in wind speed observations. In particular, the estimated shape parameters indicate moderate asymmetry and distributional flexibility, while the scale parameter reflects the overall magnitude of the observed process. These estimated values were subsequently used as the baseline distributional inputs for constructing the Phase I reference distribution and implementing the proposed Entropy–EWMA monitoring procedure under progressively Type-II censored observations.

Table 11 summarizes the numerical optimization results for fitting the Exponentiated Weibull distribution. The convergence code of zero indicates successful termination of the optimization procedure, which was achieved after 10 iterations. Together with the parameter estimates reported in Table 9, these results confirm successful numerical estimation of the baseline distributional model used in the subsequent monitoring analysis.

### 5.2. Comparative Monitoring Performance Under Progressive Type-II Censoring

The auxiliary shift-estimation smoothing parameter Ψ was fixed at 0.25 based on the simulation findings, which provided a reasonable balance between monitoring stability and responsiveness to entropy departures. To maintain full consistency between the simulation study and the real-data application, the two-sided control limits calibrated under the corresponding censoring configurations in the simulation study were retained. Accordingly, the control limit was set to *h* = 0.7025 for the Uniform censoring scheme and h = 0.6550 for the Late censoring scheme, corresponding to *n* = 100, *m* = 30, and Ψ=0.25. These limits were calibrated under in-control conditions to achieve the nominal $ARL_{0}\approx 370$. An out-of-control signal was generated whenever $\left|W_{t}\right|>h$. Thus, the real-data application employs the same two-sided monitoring rule and control-limit calibration used in the simulation study.

To evaluate practical monitoring behavior, the Classical EWMA and the proposed Entropy–EWMA procedures were applied to the Wind Turbine SCADA data under both Uniform and Late Progressive Type-II censoring schemes. The Classical EWMA monitors standardized changes in process location, whereas the proposed procedure monitors standardized entropy departures through the adaptive mechanism described in Section 3.2. For each monitoring epoch, the estimated persistent entropy-deviation magnitude $\delta_{t}$ determines the adaptive smoothing coefficient ${\lambda (\delta }_{t})$, which is subsequently used to update the Entropy–EWMA plotting statistic $W_{t}$. Monitoring performance was summarized in terms of the first detected signal, IC alarms, OOC detections, and signal direction.

Table 12 presents the comparative monitoring results under the two censoring schemes. The first 30 monitoring epochs constitute the IC phase, whereas epochs 31–50 constitute the OOC phase. IC and OOC signals are reported separately to distinguish false alarms from detections occurring after the transition point $t_{0}=30$.

Under both censoring structures, neither method produced false alarms during the IC phase (samples 1–30), indicating stable in-control monitoring behavior. Following the first detected signal, the corresponding monitoring statistic remained beyond the control limit throughout the remaining OOC epochs in each configuration. Consequently, the reported OOC alarm counts are fully consistent with the first-signal locations and the 20-epoch OOC monitoring period. Under the Uniform censoring scheme, the Classical EWMA generated its first signal at sample 33 and produced 18 OOC alarms, whereas the proposed Entropy–EWMA first signaled at sample 36 and generated 15 OOC alarms. All detected departures occurred through the upper control limit. Under the Late censoring scheme, the Classical EWMA again generated its first signal at sample 33 and produced 18 OOC alarms, while the proposed Entropy–EWMA first signaled at sample 38 and generated 13 OOC alarms. The detected departures were again in the upper direction.

The results show that the two monitoring procedures respond differently to the observed process change. The Classical EWMA, which is based on the standardized sample mean, detects the transition somewhat earlier under both censoring schemes, reflecting its direct sensitivity to changes in process location. In contrast, the proposed Entropy–EWMA responds to changes in the information structure of the progressively censored process and exhibits a more selective signaling pattern. In particular, the smaller number of OOC alarms produced by the entropy-based procedure indicates that changes in process location are not necessarily accompanied by equally strong changes in entropy.

These findings support interpreting the Classical EWMA and the proposed Entropy–EWMA as complementary rather than directly competing monitoring procedures. While the Classical EWMA primarily captures changes in process location, the Entropy–EWMA provides additional information about changes in process uncertainty and information structure. The absence of IC alarms for both procedures, together with their successful detection of post-transition changes under both censoring schemes, demonstrates stable monitoring behavior in the considered real-data application.

**Figure 7:**
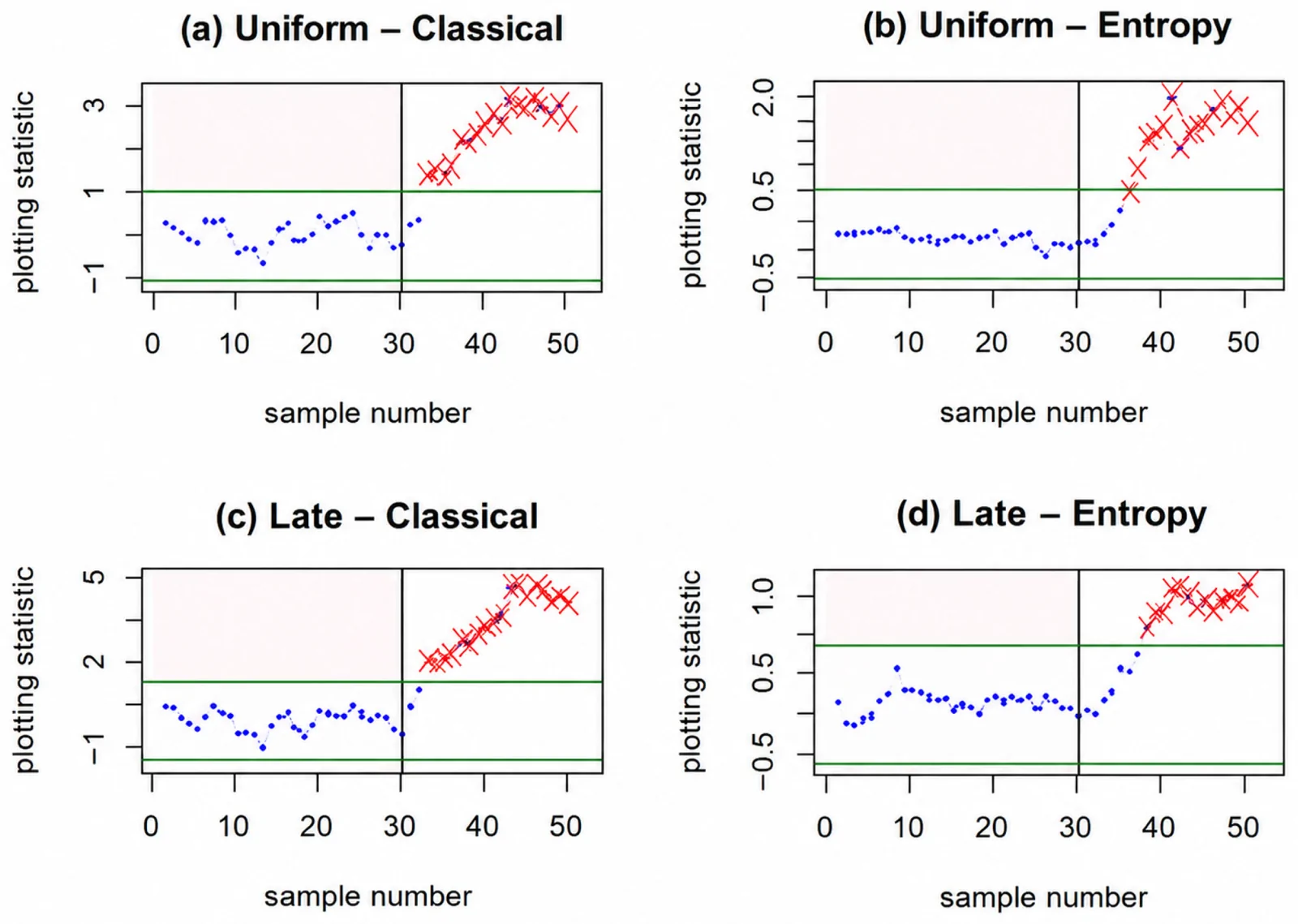
Comparison of Classical EWMA and Proposed Entropy–EWMA monitoring schemes under Uniform and Late Progressive Type-II censoring structures for the Wind Turbine SCADA data using two-sided control limits. Note: Vertical scales are shown separately for each panel to facilitate visualization and should not be interpreted as a common scale across monitoring statistics.

Figure 7 illustrates the monitoring trajectories of the Classical EWMA and proposed Entropy–EWMA procedures under the Uniform and Late censoring schemes. Under Uniform censoring, both monitoring statistics move upward after the transition point, with the Classical EWMA generating its first signal at sample 33 and the Entropy–EWMA at sample 36. A similar pattern is observed under Late censoring, where the first signals occur at samples 33 and 38, respectively. In both censoring schemes, the Entropy–EWMA signals are generated through the upper control limit, indicating an increase in the monitored entropy structure during the OOC phase. Importantly, none of the four monitoring configurations produces a signal during the first 30 IC samples, supporting stable in-control behavior.

Overall, the real-data application demonstrates that the proposed Entropy–EWMA framework provides a complementary information-theoretic perspective for monitoring progressively Type-II censored observations. The Classical EWMA responds more rapidly to the observed location change, whereas the Entropy–EWMA identifies the accompanying change in process uncertainty and information structure through its adaptive monitoring mechanism. Thus, the proposed procedure is not intended to replace conventional location-based monitoring, but rather to provide additional information on distributional changes that may not be fully characterized by changes in process location alone.

## 6. Conclusions

This study developed an Entropy–EWMA monitoring framework for detecting changes in the information structure of progressively Type-II censored lifetime processes. Unlike conventional monitoring approaches that primarily focus on changes in process location, scale, parameters, or selected quantiles, the proposed methodology monitors variations in overall uncertainty through a censoring-adjusted entropy statistic. The framework was implemented under the Exponentiated Weibull distribution, while the underlying monitoring principle is not inherently restricted to this model and can, in principle, be extended to other continuous lifetime distributions for which the corresponding density and entropy measure can be evaluated.

To account for information loss under Progressive Type-II censoring, a weighted entropy estimator was incorporated into a sequential adaptive EWMA structure. At each monitoring epoch, the Exponentiated Weibull parameters are estimated from the current progressively censored sample, the adjusted entropy statistic is standardized relative to the Phase I information baseline, and an auxiliary smoothed estimate of the persistent entropy departure is used to determine the adaptive EWMA response. This structure enables the monitoring procedure to respond to sustained changes in process uncertainty while maintaining stable in-control behavior.

The Monte Carlo simulation study demonstrated that the proposed Entropy–EWMA scheme maintains in-control ARL values close to the nominal target of ${ARL}_{0}=370$ under different censoring structures, observed sample sizes, and auxiliary shift-estimation smoothing parameter settings. More importantly, the out-of-control ARL decreased substantially as the imposed entropy degradation increased, demonstrating progressively faster detection of information-structural departures. The results also showed that monitoring performance depends on the interaction among the censoring structure, the amount of information retained, and the auxiliary smoothing setting rather than on a single universally optimal configuration. The run-length variability and quantile results further supported the ability of the proposed procedure to distinguish in-control behavior from moderate and severe entropy degradation.

The comparative simulation analysis with the Classical EWMA further demonstrated the value of entropy-based monitoring. Under comparable in-control conditions, the Entropy–EWMA scheme exhibited substantially shorter run lengths under moderate and severe entropy degradation scenarios. This finding reflects the different objectives of the two monitoring approaches: the Classical EWMA primarily responds to changes represented through conventional process statistics, whereas the proposed framework is specifically designed to detect departures in the information and uncertainty structure of the monitored process.

The practical applicability of the proposed methodology was further investigated using Wind Turbine SCADA data under Uniform and Late Progressive Type-II censoring schemes. The Exponentiated Weibull distribution was evaluated against alternative lifetime models before being used as the baseline distributional framework. In the subsequent monitoring analysis, neither the Classical EWMA nor the Entropy–EWMA produced false alarms during the in-control phase. During the out-of-control phase, both approaches generated upper-control-limit signals, but with different detection patterns. The Classical EWMA produced earlier and more persistent signals associated with changes in process location, whereas the Entropy–EWMA responded to changes in the uncertainty structure represented by the adjusted entropy statistic. These findings support interpreting the two approaches as complementary rather than competing monitoring tools.

Overall, the proposed Entropy–EWMA framework extends statistical process monitoring for progressively censored lifetime data by introducing an information-theoretic perspective that complements conventional location- and parameter-based approaches. Its principal advantage lies in summarizing distribution-level uncertainty within a single monitoring quantity while explicitly accounting for information loss induced by Progressive Type-II censoring. Nevertheless, the present study is limited to univariate monitoring, selected censoring structures, and a specific adaptive smoothing mechanism. Future research may therefore investigate multivariate extensions, alternative entropy and information measures, additional censoring mechanisms, alternative lifetime distributions, and Bayesian or distribution-free monitoring structures. A joint monitoring framework that simultaneously tracks conventional process characteristics and entropy-based information changes also represents a promising direction for future research.

## Figures and Tables

**Table 1:** Progressive Type-II censoring schemes used in the simulation study.

*n*	*m*	Scheme	Censoring Vector ${R=(R}_{1},R_{2},\ldots ,R_{m})$
100	20	Uniform	$\left(4^{\left(20\right)}\right)$
		Late	$\left(0^{\left(19\right)},80\right)$
	30	Uniform	$\left(3^{\left(10\right)},{(2}^{\left(20\right)}\right)$
		Late	$\left(0^{\left(29\right)},70\right)$
	40	Uniform	$\left(2^{\left(20\right)},{(1}^{\left(20\right)}\right)$
		Late	$\left(0^{\left(39\right)},60\right)$

**Table 2:** Run length performance of the proposed Entropy–EWMA monitoring scheme under Uniform and Late Progressive Type-II censoring structures Ψ=0.05, n=100.

Scheme	*m*	$\Delta_{H}$	*h*	${\hat{\mu }}_{H}$	${\hat{\sigma }}_{H}$	*ARL*	SDRL	$Q_{RL}$ (0.05)	$Q_{RL}$ (0.25)	$Q_{RL}$ (0.50)	$Q_{RL}$ (0.75)
Uniform	20	0.00	0.4925	0.6781	0.1729	354.34	401.81	3.00	57.00	221.00	518.00
		0.50	0.4925	0.6781	0.1729	17.89	15.71	2.00	6.00	14.00	25.00
		1.50	0.4925	0.6781	0.1729	3.31	1.70	2.00	2.00	3.00	4.00
	30	0.00	0.5275	0.6986	0.1311	358.19	427.65	3.00	55.75	221.00	507.00
		0.50	0.5275	0.6986	0.1311	19.27	17.65	2.00	6.00	15.00	26.00
		1.50	0.5275	0.6986	0.1311	3.41	1.79	2.00	2.00	3.00	4.00
	40	0.00	0.5100	0.7260	0.1171	357.05	402.57	3.00	52.00	224.50	512.00
		0.50	0.5100	0.7260	0.1171	18.92	16.80	2.00	6.00	15.00	26.00
		1.50	0.5100	0.7260	0.1171	3.34	1.73	2.00	2.00	3.00	4.00
Late	20	0.00	0.4800	0.5042	0.2804	381.57	430.74	3.00	64.00	248.00	541.00
		0.50	0.4800	0.5042	0.2804	17.63	15.01	2.00	6.00	14.00	24.00
		1.50	0.4800	0.5042	0.2804	3.20	1.62	2.00	2.00	3.00	4.00
	30	0.00	0.4975	0.5099	0.2270	369.63	427.47	3.00	59.75	218.00	533.00
		0.50	0.4975	0.5099	0.2270	17.84	15.45	2.00	6.00	14.00	24.00
		1.50	0.4975	0.5099	0.2270	3.31	1.71	2.00	2.00	3.00	4.00
	40	0.00	0.5375	0.5495	0.1906	371.60	417.57	3.00	64.00	235.00	528.00
		0.50	0.5375	0.5495	0.1906	25.49	24.26	2.00	8.00	19.00	35.00
		1.50	0.5375	0.5495	0.1906	3.74	2.12	2.00	2.00	3.00	5.00

**Table 3:** Run length performance of the proposed Entropy–EWMA monitoring scheme under Uniform and Late Progressive Type-II censoring structures Ψ=0.10, n=100.

Scheme	*m*	$\Delta_{H}$	*h*	${\hat{\mu }}_{H}$	${\hat{\sigma }}_{H}$	*ARL*	SDRL	$Q_{RL}$ (0.05)	$Q_{RL}$ (0.25)	$Q_{RL}$ (0.50)	$Q_{RL}$ (0.75)
Uniform	20	0.00	0.5400	0.6781	0.1729	374.08	401.58	4.00	82.00	249.00	526.00
		0.50	0.5400	0.6781	0.1729	21.43	19.14	2.00	7.00	17.00	29.00
		1.50	0.5400	0.6781	0.1729	3.47	1.84	2.00	2.00	3.00	4.00
	30	0.00	0.5775	0.6986	0.1311	377.82	395.32	4.00	87.00	258.00	534.00
		0.50	0.5775	0.6986	0.1311	23.00	20.15	2.00	7.00	18.00	32.00
		1.50	0.5775	0.6986	0.1311	3.58	1.99	2.00	2.00	3.00	4.00
	40	0.00	0.5550	0.7260	0.1171	363.54	390.91	3.00	81.00	244.00	512.00
		0.50	0.5550	0.7260	0.1171	21.84	19.58	2.00	7.00	17.00	30.00
		1.50	0.5550	0.7260	0.1171	3.51	1.86	2.00	2.00	3.00	4.00
Late	20	0.00	0.5250	0.5042	0.2804	393.36	423.04	4.95	91.75	268.00	546.25
		0.50	0.5250	0.5042	0.2804	19.77	16.82	2.00	7.00	16.00	28.00
		1.50	0.5250	0.5042	0.2804	3.30	1.66	2.00	2.00	3.00	4.00
	30	0.00	0.5425	0.5099	0.2270	358.82	381.60	4.00	79.00	238.00	511.00
		0.50	0.5425	0.5099	0.2270	20.60	17.48	2.00	7.00	16.00	29.00
		1.50	0.5425	0.5099	0.2270	3.43	1.78	2.00	2.00	3.00	4.00
	40	0.00	0.5800	0.5495	0.1906	368.54	401.80	4.00	83.00	241.00	519.25
		0.50	0.5800	0.5495	0.1906	29.83	26.53	2.00	9.00	23.00	42.00
		1.50	0.5800	0.5495	0.1906	3.80	2.20	2.00	2.00	3.00	5.00

**Table 4:** Run length performance of the proposed Entropy–EWMA monitoring scheme under Uniform and Late Progressive Type-II censoring structures Ψ=0.25, n=100.

Scheme	*m*	$\Delta_{H}$	*h*	${\hat{\mu }}_{H}$	${\hat{\sigma }}_{H}$	*ARL*	SDRL	$Q_{RL}$ (0.05)	$Q_{RL}$ (0.25)	$Q_{RL}$ (0.50)	$Q_{RL}$ (0.75)
Uniform	20	0.00	0.6450	0.6781	0.1729	369.50	379.05	8.00	95.00	246.50	528.25
		0.50	0.6450	0.6781	0.1729	28.20	25.49	3.00	10.00	21.00	39.00
		1.50	0.6450	0.6781	0.1729	3.79	2.04	2.00	2.00	3.00	5.00
	30	0.00	0.7025	0.6986	0.1311	359.05	376.31	9.00	94.00	241.00	501.00
		0.50	0.7025	0.6986	0.1311	30.13	27.65	3.00	10.00	22.00	42.00
		1.50	0.7025	0.6986	0.1311	4.08	2.28	2.00	2.00	3.00	5.00
	40	0.00	0.6750	0.7260	0.1171	368.54	376.84	8.00	102.00	252.00	514.00
		0.50	0.6750	0.7260	0.1171	30.06	27.60	3.00	10.00	22.00	41.00
		1.50	0.6750	0.7260	0.1171	3.98	2.20	2.00	2.00	3.00	5.00
Late	20	0.00	0.6150	0.5042	0.2804	363.91	370.46	8.00	95.00	253.00	523.25
		0.50	0.6150	0.5042	0.2804	26.41	23.38	3.00	9.00	20.00	36.00
		1.50	0.6150	0.5042	0.2804	3.66	1.92	2.00	2.00	3.00	4.00
	30	0.00	0.6550	0.5099	0.2270	373.97	386.56	9.00	98.00	255.00	525.25
		0.50	0.6550	0.5099	0.2270	27.11	25.11	3.00	9.00	20.00	37.00
		1.50	0.6550	0.5099	0.2270	3.87	2.11	2.00	2.00	3.00	5.00
	40	0.00	0.7100	0.5495	0.1906	374.48	379.33	9.00	98.00	257.00	526.25
		0.50	0.7100	0.5495	0.1906	40.93	39.01	3.00	13.00	29.00	56.00
		1.50	0.7100	0.5495	0.1906	4.35	2.55	2.00	2.00	4.00	6.00

**Table 5:** Run length performance of the proposed Entropy–EWMA monitoring scheme under Uniform and Late Progressive Type-II censoring structures Ψ=0.50, n=100.

Scheme	*m*	$\Delta_{H}$	*h*	${\hat{\mu }}_{H}$	${\hat{\sigma }}_{H}$	*ARL*	SDRL	$Q_{RL}$ (0.05)	$Q_{RL}$ (0.25)	$Q_{RL}$ (0.50)	$Q_{RL}$ (0.75)
Uniform	20	0.00	0.7875	0.6781	0.1729	380.88	371.49	18.00	108.00	267.00	537.00
		0.50	0.7875	0.6781	0.1729	35.82	31.61	4.00	13.00	27.00	49.00
		1.50	0.7875	0.6781	0.1729	4.40	2.50	2.00	3.00	4.00	6.00
	30	0.00	0.8600	0.6986	0.1311	368.64	370.01	16.00	104.00	254.00	513.25
		0.50	0.8600	0.6986	0.1311	41.83	37.95	4.00	15.00	31.00	56.00
		1.50	0.8600	0.6986	0.1311	4.69	2.81	2.00	3.00	4.00	6.00
	40	0.00	0.8175	0.7260	0.1171	367.85	371.19	15.00	101.00	251.00	515.00
		0.50	0.8175	0.7260	0.1171	38.42	34.78	4.00	14.00	28.00	52.00
		1.50	0.8175	0.7260	0.1171	4.52	2.62	2.00	3.00	4.00	6.00
Late	20	0.00	0.7550	0.5042	0.2804	368.76	376.59	18.00	100.00	254.00	509.00
		0.50	0.7550	0.5042	0.2804	33.07	28.90	4.00	13.00	25.00	45.00
		1.50	0.7550	0.5042	0.2804	4.19	2.28	2.00	2.00.00	4.00	5.00
	30	0.00	0.7925	0.5099	0.2270	374.42	375.97	16.00	107.00	260.00	520.00
		0.50	0.7925	0.5099	0.2270	35.64	31.06	4.00	13.00	27.00	48.00
		1.50	0.7925	0.5099	0.2270	4.39	2.48	2.00	3.00	4.00	6.00
	40	0.00	0.8400	0.5495	0.1906	359.96	366.20	16.00	96.00	246.00	505.00
		0.50	0.8400	0.5495	0.1906	50.87	48.19	4.00	17.00	37.00	69.00
		1.50	0.8400	0.5495	0.1906	5.03	3.01	2.00	3.00	4.00	6.00

**Table 6:** Run length performance of the proposed Entropy–EWMA monitoring scheme under Uniform and Late Progressive Type-II censoring structures Ψ=0.80,  n=100.

Scheme	*m*	$\Delta_{H}$	*h*	${\hat{\mu }}_{H}$	${\hat{\sigma }}_{H}$	*ARL*	SDRL	$Q_{RL}$ (0.05)	$Q_{RL}$ (0.25)	$Q_{RL}$ (0.50)	$Q_{RL}$ (0.75)
Uniform	20	0.00	0.9975	0.6781	0.1729	366.01	360.59	22.00	107.00	255.50	505.25
		0.50	0.9975	0.6781	0.1729	52.44	47.89	6.00	18.00	38.00	72.00
		1.50	0.9975	0.6781	0.1729	5.26	3.17	2.00	3.00	4.00	7.00
	30	0.00	1.1100	0.6986	0.1311	368.09	370.92	20.00	107.00	256.00	506
		0.50	1.1100	0.6986	0.1311	64.74	61.70	7.00	22.00	46.00	87.00
		1.50	1.1100	0.6986	0.1311	5.86	3.65	2.00	3.00	5.00	8.00
	40	0.00	1.0400	0.7260	0.1171	361.25	361.36	20.00	105.00	249.00	500.25
		0.50	1.0400	0.7260	0.1171	54.61	50.05	6.00	19.00	40.00	74.00
		1.50	1.0400	0.7260	0.1171	5.51	3.25	2.00	3.00	5.00	7.00
Late	20	0.00	0.9575	0.5042	0.2804	367.42	365.66	22.00	111.00	258.00	512.00
		0.50	0.9575	0.5042	0.2804	47.89	44.15	5.95	17.00	35.00	64.00
		1.50	0.9575	0.5042	0.2804	5.06	2.86	2.00	3.00	4.00	6.00
	30	0.00	0.9975	0.5099	0.2270	371.47	362.01	21.00	112.75	261.50	518.00
		0.50	0.9975	0.5099	0.2270	48.83	44.05	6.00	18.00	35.00	67.00
		1.50	0.9975	0.5099	0.2270	5.24	3.11	2.00	3.00	4.00	7.00
	40	0.00	1.0600	0.5495	0.1906	369.80	365.79	22.00	106.00	257.00	515.00
		0.50	1.0600	0.5495	0.1906	68.74	64.33	7.00	23.00	50.00	94.00
		1.50	1.0600	0.5495	0.1906	6.12	3.98	2.00	3.00	5.00	8.00

**Table 7:** Comparative run-length performance of the classical EWMA and proposed Entropy–EWMA charts under entropy degradation scenarios (*n* = 100, *m* = 30, Ψ=0.25).

Scheme	$\Delta_{H}$	Classical ARL	Classical SDRL	Entropy ARL	Entropy SDRL	Reduction (%)
Uniform	0.00	361.54	354.76	359.05	376.31	0.69
	0.50	363.35	349.97	30.13	27.65	91.71
	1.50	360.57	360.47	4.08	2.28	98.87
Late	0.00	378.06	372.98	373.97	386.56	1.08
	0.50	380.36	369.63	27.11	25.11	92.87
	1.50	374.77	363.52	3.87	2.11	98.97

**Table 8:** Summary statistics of Wind Turbine SCADA dataset (Wind Speed, m/s).

Statistic	Value
Sample Size (n)	50,520
Minimum	0.00
Maximum	25.2060
Mean	7.5594
Variance	17.8612
Standard Deviation	4.2262
Coefficient of Variation	0.5591
Skewness	0.3838
Kurtosis	3.0588

**Table 9:** Goodness-of-fit comparison of alternative lifetime distributions fitted to the Wind Turbine SCADA data.

Measure	Exponentiated Weibull	Weibull	Generalized Gamma
Log-Likelihood	−141,001.1461	−141,022.2667	−141,003.6453
AIC	282,008.2923	282,048.5334	282,013.2907
BIC	282,034.7827	282,066.1936	282,039.7810
KS statistic (D)	0.0202	0.0222	0.0204
Cramér–von Mises (W)	3.1609	5.1620	3.2516
Anderson–Darling (A)	23.1850	33.6011	23.7187

**Table 10:** Maximum likelihood estimates for Exponentiated Weibull distribution parameters.

Parameter	Estimate (θ′)	Standard Error (SE)	95% Lower CI	95% Upper CI
α (Shape)	0.8720	0.0181	0.8366	0.9074
β (Shape)	2.0134	0.0259	1.9627	2.0641
λ (Scale)	9.0703	0.0866	8.9005	9.2401

**Table 11:** Convergence and optimization summary for Exponentiated Weibull distribution fitting.

Metric	Value
Log-Likelihood value	−141,001.1461
Convergence code	0 (Successful)
Total iterations	10

**Table 12:** Detection Performance Comparison of Classical EWMA and Entropy–EWMA: First Signal, False Alarm Control, and Signal Direction under Uniform and Late Progressive Type-II Censoring.

Scheme	Method	First Signal	IC Alarms	OOC Alarms	Signal Direction
Uniform	Classical EWMA	33	0	18	Upper
	Proposed Entropy–EWMA	36	0	15	Upper
Late	Classical EWMA	33	0	18	Upper
	Proposed Entropy–EWMA	38	0	13	Upper

## Data Availability

Data are contained within the article. The data used in the study have been cited.

## Appendix A

**Table A1:** Summary of the main notation used in the proposed Entropy–EWMA framework.

n	Initial number of units placed on test
m	Number of observed failures under Progressive Type-II censoring
$R_{i}$	Number of surviving units removed immediately after the i*th* observed failure
$w_{i}$	Normalized stage-representation weight for the i*th* censoring stage
α,β,λ	Shape, shape, and scale parameters of the Exponentiated Weibull distribution, respectively
t	Monitoring epoch
${\hat{\alpha }}_{t},{\hat{\beta }}_{t},{\hat{\lambda }}_{t}$	Maximum likelihood estimates obtained from the censored sample at monitoring epoch t
${\hat{H}}_{t}^{adj}$	Censoring-adjusted entropy estimate at monitoring epoch t
D	Number of independent in-control samples used for Phase I calibration
$\mu_{H}$	Phase I reference mean of the adjusted entropy statistic
$\sigma_{H}$	Phase I reference standard deviation of the adjusted entropy statistic
$Z_{t}$	Standardized adjusted-entropy deviation at monitoring epoch t
$\Delta_{H}$	True entropy-degradation magnitude imposed in the simulation study
Ψ	Auxiliary shift-estimation smoothing parameter
$\delta_{t}^{*}$	Smoothed auxiliary entropy-deviation estimator
$\delta_{t}^{**}$	Initialization-corrected auxiliary entropy-deviation estimator
${\hat{\delta }}_{t}$	Estimated magnitude of the persistent standardized entropy departure
${\lambda (\hat{\delta }}_{t*1})$	Adaptive EWMA smoothing coefficient used at monitoring epoch t
$W_{t}$	Entropy–EWMA plotting statistic at monitoring epoch t
h	Monte Carlo-calibrated two-sided control limit
RL	Run length; number of monitoring epochs until the first signal
ARL	Average Run Length
SDRL	Standard deviation of Run Length
$Q_{RL}(p)$	p*th* quantile of the run-length distribution
